# Atypical UV Photoproducts Induce Non-canonical Mutation Classes Associated with Driver Mutations in Melanoma

**DOI:** 10.1016/j.celrep.2020.108401

**Published:** 2020-11-17

**Authors:** Marian F. Laughery, Alexander J. Brown, Kaitlynne A. Bohm, Smitha Sivapragasam, Haley S. Morris, Mila Tchmola, Angelica D. Washington, Debra Mitchell, Stephen Mather, Ewa P. Malc, Piotr A. Mieczkowski, Steven A. Roberts, John J. Wyrick

**Affiliations:** 1 School of Molecular Biosciences, Washington State University, Pullman, WA 99164, USA; 2 Center for Reproductive Biology, Washington State University, Pullman, WA 99164, USA; 3 Department of Genetics, Lineberger Comprehensive Cancer Center, University of North Carolina, Chapel Hill, NC 27599, USA; 4 Present address: AbSci, Vancouver, WA 98660, USA; 5 Present address: Seattle Genetics, Bothell, WA 98021, USA; 6 Present address: Department of Mathematics, University of Tennessee, Knoxville, Knoxville, TN 37996, USA; 7 Lead Contact

## Abstract

Somatic mutations in skin cancers and other ultraviolet (UV)-exposed cells are typified by C>T and CC>TT substitutions at dipyrimidine sequences; however, many oncogenic “driver” mutations in melanoma do not fit this UV signature. Here, we use genome sequencing to characterize mutations in yeast repeatedly irradiated with UV light. Analysis of ~50,000 UV-induced mutations reveals abundant non-canonical mutations, including T>C, T>A, and AC>TT substitutions. These mutations display transcriptional asymmetry that is modulated by nucleotide excision repair (NER), indicating that they are caused by UV photoproducts. Using a sequencing method called UV DNA endonuclease sequencing (UVDE-seq), we confirm the existence of an atypical thymine-adenine photoproduct likely responsible for UV-induced T>A substitutions. Similar non-canonical mutations are present in skin cancers, which also display transcriptional asymmetry and dependence on NER. These include multiple driver mutations, most prominently the recurrent *BRAF* V600E and V600K substitutions, suggesting that mutations arising from rare, atypical UV photoproducts may play a role in melanomagenesis.

## In Brief

UV mutagenesis has been well studied, but many driver mutations in melanoma do not fit the canonical UV signature. Using whole-genome sequencing, Laughery et al. show that UV induces a broader spectrum of mutations than anticipated. Non-canonical UV mutations are likely caused by atypical photoproducts, which may contribute to melanomagenesis.

## Graphical Abstract

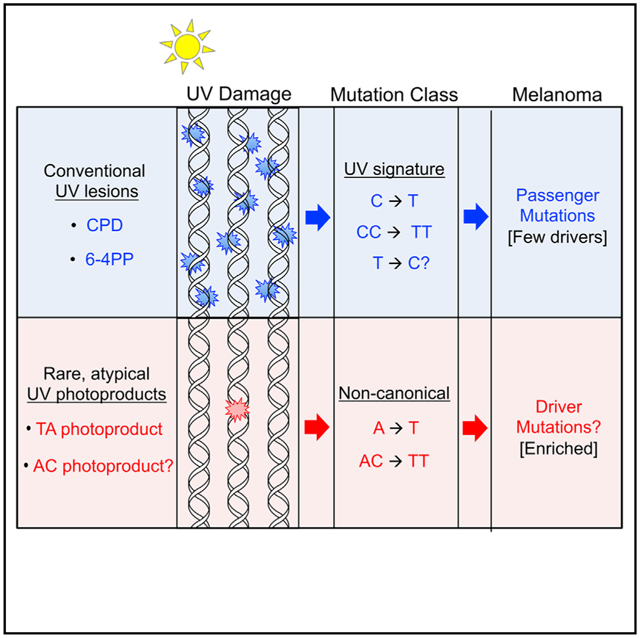

## INTRODUCTION

Exposure to ultraviolet (UV) light causes a unique signature of mutations in skin cancers and other UV-irradiated cells (Brash, 2015; Ikehata and Ono, 2011; Pfeifer et al., 2005). UV-induced mutations primarily consist of C-to-T (C>T) substitutions in cytosine-containing dipyrimidine (Dipyr) sequences (i.e., TC, CT, or CC). Tandem C>T mutations (i.e., CC>TT) are also enriched in UV-exposed cells, although these occur less frequently than single C>T substitutions. Hence CC>TT and C>T substitutions in Dipyr sequences comprise the canonical signature of short- or medium-wavelength UV light (i.e., UVC or UVB) (Brash, 2015). UV signature mutations arise from mutagenic bypass of UV-induced DNA lesions, primarily consisting of cyclobutane pyrimidine dimers (CPDs) and (6–4) photoproducts (6–4PPs) (Ikehata and Ono, 2011; Pfeifer and Besaratinia, 2012), which form exclusively at Dipyr sequences (Friedberg et al., 2006).

Although the vast majority of somatic mutations in skin cancers such as melanoma are UV signature mutations, many of the identified driver mutations in melanoma are not (Hodis et al., 2012; Pandiani et al., 2017; Sample and He, 2018). For example, two of the most frequent driver mutations in melanoma are the *NRAS* Q61R and the *BRAF* V600E mutations. Although these are among the most recurrent mutations in melanoma and are associated with carcinogenesis, neither is a canonical UV signature mutation: *NRAS* Q61R is caused by a T>C mutation, while *BRAF* V600E is caused by a T>A mutation in a non-Dipyr context (Hodis et al., 2012; Pandiani et al., 2017; Sample and He, 2018). Similarly, the most recurrent tandem mutation in a melanoma driver gene is an AC>TT substitution that causes the *BRAF* V600K mutation (Hayward et al., 2017; Menzies et al., 2012; Rubinstein et al., 2010; Thomas et al., 2004). Because this tandem mutation involves a non-Dipyr sequence (i.e., AC), it also does not fit the known UV signature. As a whole, fewer than 50% of putative driver mutations in melanoma are UV signature mutations (Hodis et al., 2012), which is surprising given the known association between acute UV exposure (i.e., blistering sunburns) and melanomagenesis (Garibyan and Fisher, 2010). Genome sequencing of mutations arising in mammalian cells following experimental UV exposure (typically a single low dose of UV) has confirmed that UVB or UVC light primarily induces UV signature mutations (Kucab et al., 2019; Nik-Zainal et al., 2015; Olivier et al., 2014). However, these studies have not provided insight into the origin of the atypical substitution patterns that cause many of the driver mutations in melanoma. Non-UV signature driver mutations in *BRAF* (and potentially other genes) could arise from a neighboring UV lesion (Thomas et al., 2006), but support for this hypothesis has been difficult to ascertain given the limited numbers of non-UV signature mutations in melanoma and other UV-exposed cells, and because of the difficulty in establishing their UV origin.

Mutations arising from UV photoproducts possess two distinguishing characteristics. First, these mutations are elevated in cells defective in the nucleotide excision repair (NER) pathway, which is required to repair bulky UV photoproducts and other helix-distorting DNA lesions (Schärer, 2013). For example, genetic defects in the NER pathway in xeroderma pigmentosum (XP) patients cause elevated frequency of UV mutations (Zheng et al., 2014), which translates to a >1,000-fold increased risk for skin cancer (DiGiovanna and Kraemer, 2012). Second, UV signature mutations are depleted from the transcribed strand (TS) of expressed genes, a characteristic that is termed “transcriptional asymmetry” (Haradhvala et al., 2016). This transcriptional asymmetry is due to more efficient repair of UV photoproducts along the TS by the NER sub-pathway known as transcription-coupled-NER (TC-NER). TC-NER operates only on the TS of expressed genes, because it is initiated when RNA polymerase stalls at UV photoproducts (Geijer and Marteijn, 2018; Hanawalt and Spivak, 2008). Genome sequencing of cutaneous squamous cell carcinomas (cSCC) and melanomas has revealed that UV signature mutations have significant transcriptional asymmetry (Haradhvala et al., 2016; Pleasance et al., 2010; Zheng et al., 2014). This asymmetry is elevated in tumors derived from individuals with germline deficiencies in the *XPC* gene (Zheng et al., 2014), because *XPC* is required for the global genomic-NER (GG-NER) sub-pathway, which repairs UV photoproducts in intergenic DNA and the non-transcribed strand (NTS) of genes.

Here, we exploited these characteristics of UV-induced mutations to identify novel mutation classes arising from exposure to UV light. We sequenced the genomes of >150 independent isolates of wild-type (WT) or NER-deficient yeast strains and identified in total more than 50,000 UV-induced mutations. Although canonical UV signature mutations are prevalent in our dataset, we also observe other mutation classes likely associated with atypical UV lesions, including a thymine-adenine (TA) photoproduct, which we mapped at single-nucleotide resolution across UV-irradiated yeast genomes. We show that similar mutation classes occur at low abundance in cutaneous melanomas and squamous cell carcinomas, with characteristics consistent with these mutations arising from bulky UV photoproducts. These non-canonical mutation classes include *NRAS* Q61R and *BRAF* V600K and V600E, which are among the most common driver mutations in melanoma, indicating that mutations caused by atypical photoproducts may promote melanomagenesis.

## RESULTS

### Genome Sequencing of UV-Exposed Yeast Reveals Novel UV-Induced Mutation Classes

**Figure 1.**
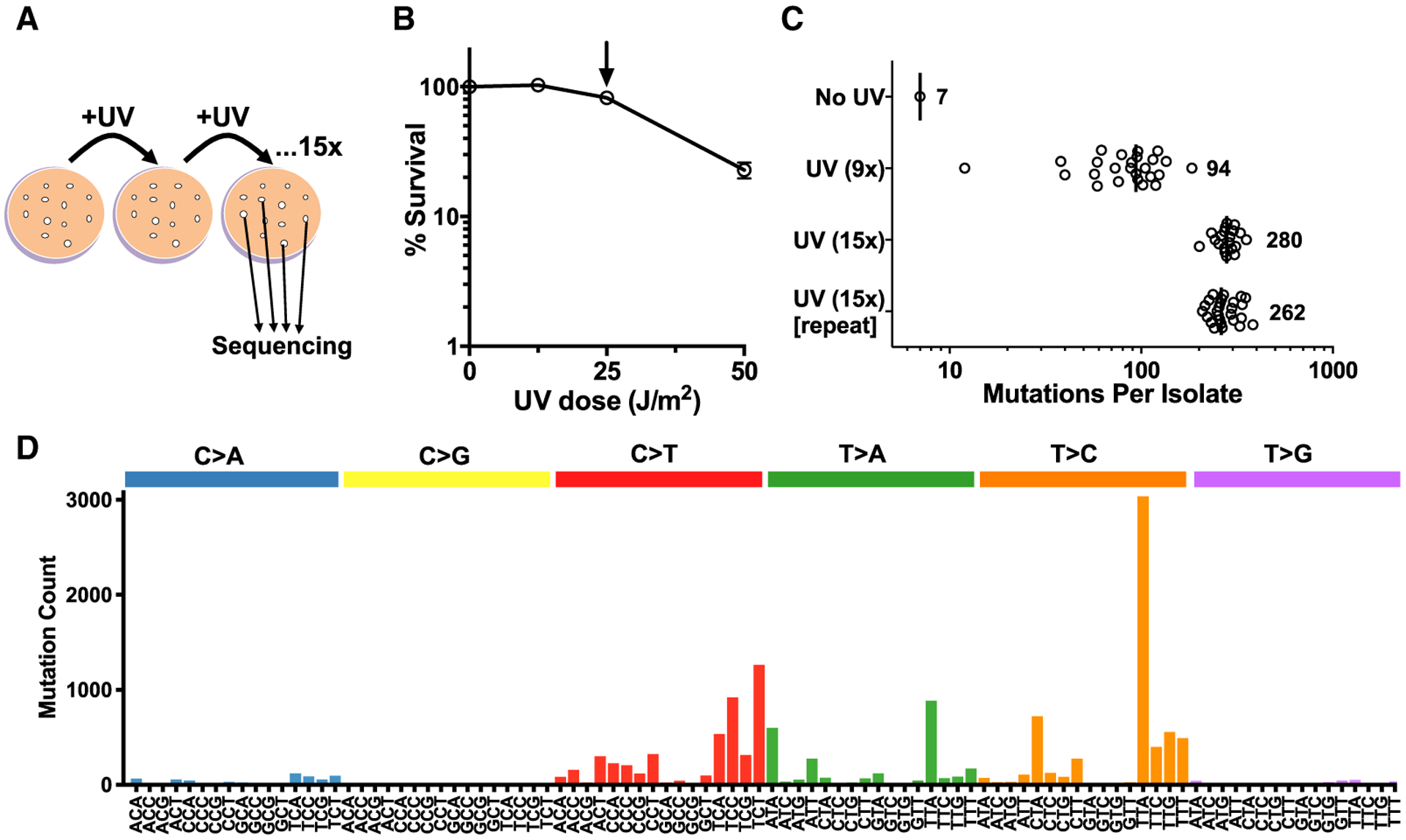
Genome Sequencing of UV-Induced Mutations in Yeast Reveals Non-canonical Mutation Signatures (A) Experimental procedure for genomic sequencing of UV-induced mutations accrued following 15 exposures to UVC light (25 J/m^2^) in independent yeast isolates. (B) Percentage of surviving cells following exposure of wild-type (WT) yeast to a single dose of UVC light. Arrow indicates the dose used for genome sequencing experiments (25 J/m^2^). (C) Number of mutations per isolate of WT accrued following 9× or 15× exposures to UVC light (25 J/m^2^). Mutations were identified by genome sequencing of each independent yeast isolate. (D) Mutation profile of single-nucleotide substitutions in UV-exposed yeast (9× and 15× doses). The mutation count for each substitution type (e.g., C>A, C>G, etc.) and trinucleotide context is depicted. The middle base of each trinucleotide context is mutated.

To better define the complete spectrum of UV-induced mutations in eukaryotic cells, we repeatedly exposed diploid yeast to either 9 or 15 doses of 25 J/m^2^ UVC light and then sequenced the genomes of individual isolates to identify UV-induced mutations (Figure 1A; Data S1). This treatment has a minor effect on WT yeast survival (Figure 1B) but induces a highly reproducible, dose-dependent increase in mutation in both whole-genome sequencing analysis (Figures 1C and S1A) and using a *CAN1* forward mutation reporter (Figure S1B). The >10-fold higher mutation density in UV-irradiated yeast compared with non-irradiated controls indicates that the vast majority of the mutations in exposed isolates are UV induced.

To discern mutations potentially caused by different UV-induced lesions among the aggregated data, we initially analyzed the trinucleotide context of single-nucleotide substitutions for UV-exposed WT cells. Of the 14,285 single-nucleotide substitutions, 32% are C>T mutations at either the 5′ position (5′ Dipyr) or 3′ position (3′ Dipyr) of Dipyr sequences, consistent with the traditional UV signature (Figures 1D, S1C, and S1D). However, we also identified similarly high levels of T>C (42%) and T>A (18%) mutations (Figure 1D), each enriched at specific trinucleotide contexts. T>C substitutions are primarily associated with TTA, TTC, TTG, and TTT sequences (i.e., TTN), as well as CTN trinucleotides (Figure 1D), and thus mostly occur in the 3′ position of a Dipyr (Figure S1D), indicating that they likely arise from known UV photoproducts (e.g., CPDs or 6–4PPs). In contrast, T>A substitutions primarily occur at ATA and TTA trinucleotide sequences (Figure 1D). The high abundance of T>A substitutions at non-Dipyr contexts (“No” in Figure S1D) suggests that these mutations may be caused by damage other than the canonical UV photoproducts.

### Non-canonical UV Mutations Are Induced by UV Photoproducts

**Figure 2.**
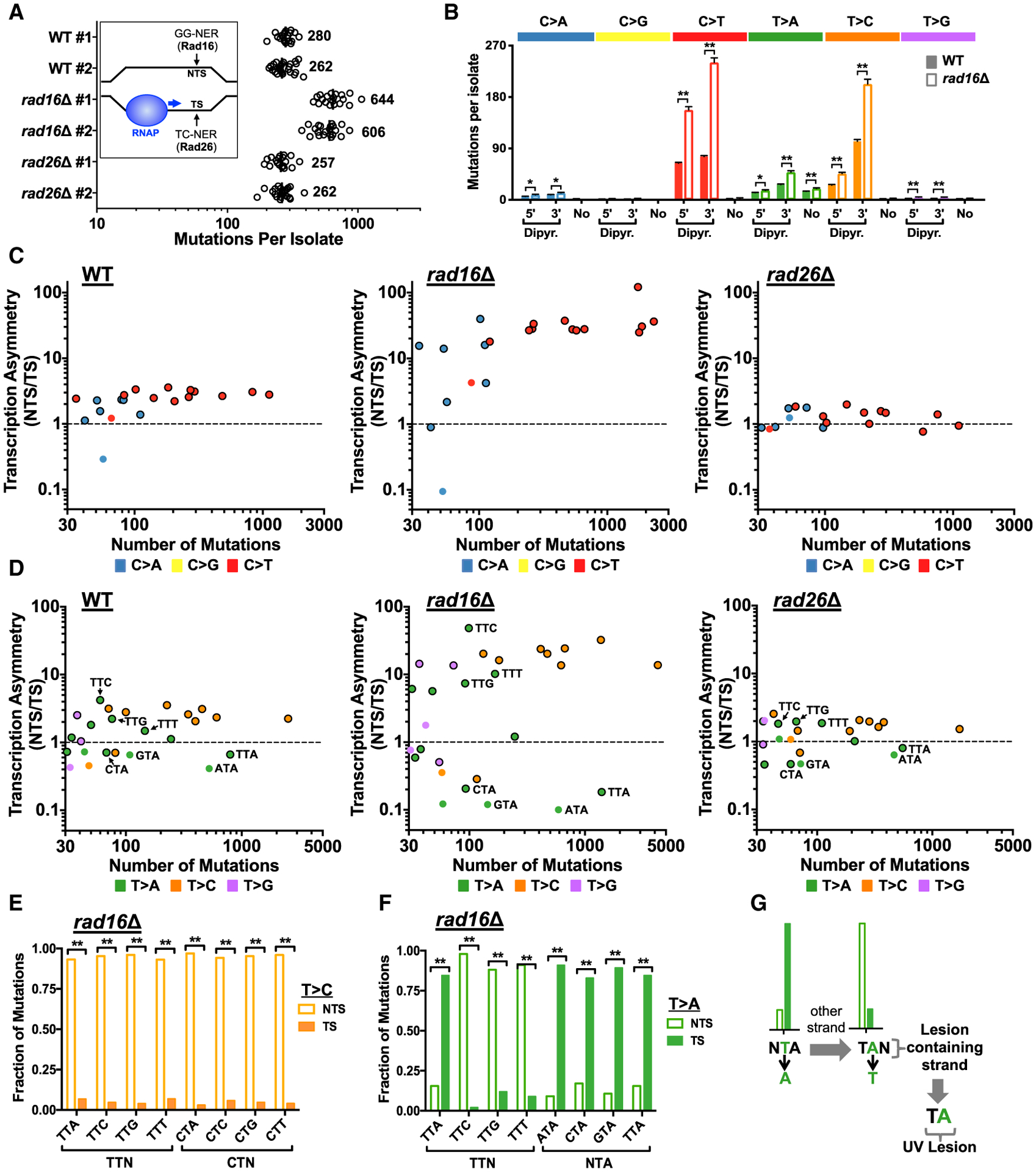
UV-Induced Mutations in Yeast Are Suppressed by NER and Show Transcriptional Asymmetry (A) Number of mutations in each genome-sequenced WT or NER-deficient isolate is plotted. Inset: schematic showing that repair of the TS by the transcription-coupled-nucleotide excision repair (TC-NER) pathway is primarily dependent upon Rad26 in yeast, while the global genomic-nucleotide excision repair (GG-NER) pathway requires Rad16. (B) Deletion of *RAD16* significantly increases the frequency of both canonical (i.e., C>T) and non-canonical (e.g., T>A or T>C) UV-induced mutations. Mutations are classified as being either in the 5′ or 3′ position of a dipyrimidine (“Dipyr”) or not in a dipyrimidine (“No”; see Figures S1C and S1D). Mean ± SEM is depicted for WT or *rad16*Δ mutant isolates. Significant differences were determined using a t test with the Holm-Sidak correction for multiple hypothesis testing. **p < 0.001; *p < 0.01. (C) Transcriptional asymmetry (i.e., normalized ratio of mutations on NTS relative to TS across all yeast genes) is plotted relative to total number of mutations for each trinucleotide context and each C>N mutation class in WT, *rad16*Δ, and *rad26*Δ mutants. Only mutation classes with at least 30 mutations are plotted. The color of the circle indicates the mutation class (e.g., C>T); mutation classes in a dipyrimidine are plotted as a circle with a black outline. (D) Same as (C), except for T>N mutation classes. (E) Fraction of T>C mutations in genes occurring on the non-transcribed strand (NTS) relative to the transcribed strand (TS) for the indicated trinucleotide contexts in *rad16*Δ mutant cells. Statistical significance was determined using the chi-square test and Bonferroni correction for multiple hypothesis testing. **p < 0.001; *p < 0.05. (F) T>A mutations are significantly enriched on the TS relative to the NTS at NTA sequences (e.g., ATA, CTA, GTA, TTA) in *rad16*Δ mutant cells. Statistical significance was determined using the chi-square test and Bonferroni correction for multiple hypothesis testing. **p < 0.001; *p < 0.05. (G) Schematic showing that elevated NTA mutations on the TS indicate that causative lesion is located on the other DNA strand (i.e., NTS) at TAN sequences (e.g., TAA, TAC, TAG, TAT).

Because mutations arising from bulky UV photoproducts are suppressed by NER, we analyzed UV-induced mutations in yeast strains defective in the GG-NER (*rad16*Δ) and TC-NER (*rad26*Δ) sub-pathways of NER (Figure 2A) to investigate whether T>C and T>A substitutions are caused by bulky UV photoproducts. The *rad16*Δ and *rad26*Δ mutant strains were treated, respectively, with 15 doses of 12.5 or 25 J/m^2^ UV light (*rad16*Δ yeast were treated with a lower dose because of their greater UV sensitivity; see Figure S2A), and the genomes of individual isolates from each strain were sequenced. Independent experimental replicates showed very similar mutation frequencies per isolate in each repair-deficient strain (Figure 2A) and nearly identical mutation spectra (Figures S2C and S2D). Consistent with single-dose mutation frequencies measured in the *CAN1* gene (Figure S2B), WT and *rad26*Δ strains displayed nearly equal numbers of mutations per genome, whereas mutations in *rad16*Δ yeast increased ~2-fold despite the lower UV dose (Figure 2A).

The *rad26*Δ cells displayed a mutation spectrum very similar to WT (Figure S2E), consistent with the limited role of Rad26 and of the TC-NER pathway in the repair of UV damage in yeast (Boiteux and Jinks-Robertson, 2013). In contrast, specific classes of mutations are elevated in the GG-NER-deficient *rad16*Δ yeast (Figure 2B). The greatest increase is observed for C>T mutations in a Dipyr context, which increase 2.5-fold (5′ Dipyr) and 3.2-fold (3′ Dipyr) in the *rad16*Δ mutant. Although these measured increases in mutation frequencies are underestimates (i.e., because of the lower dose of UV used with the *rad16*Δ mutant), these results are consistent with C>T mutations in Dipyr sequences originating from UV photoproducts that are repaired by GG-NER. Surprisingly, T>A mutations (in a 3′ Dipyr and “No” Dipyr context) and T>C mutations (5′ and 3′ Dipyr contexts) also increased in the *rad16*Δ mutant (Figure 2B), suggesting that these non-canonical UV-induced mutations may also originate from UV photoproducts repaired by the NER pathway.

To further test this hypothesis, we examined the transcriptional asymmetry of UV-induced mutations in WT and repair-deficient strains. For this analysis, we assigned the mutation to the DNA strand containing the pyrimidine base, because UV mutations are primarily associated with lesions at pyrimidine sequences. In WT cells, mutation density as a whole is ~1.9-fold lower on the TS relative to the NTS across ~5,000 yeast genes (p < 0.0001; Figure S3A), consistent with faster repair of the TS by the TC-NER pathway. Transcriptional asymmetry in mutation density is specifically associated with the transcribed region of each gene (i.e., between the transcription start site [TSS] and transcription end site [TES]) and does not significantly differ in neighboring intergenic regions (p > 0.05). Deletion of *RAD16*, which is required for GG-NER (Figure 2A, inset), increases mutation density on the NTS (Figure S3B), resulting in an elevated transcriptional asymmetry (~6.2-fold asymmetry). In contrast, deletion of *RAD26*, which plays a role in transcription-coupled repair of the TS (Figure 2A, inset), nearly eliminates the transcriptional asymmetry of UV-induced mutations (~1.2-fold asymmetry; Figure S3C).

To visualize transcriptional asymmetry for different classes of mutations, we plotted the ratio of the mutation density on the NTS relative to TS across all yeast genes for each trinucleotide mutation class (Figures 2C and 2D). As expected for canonical UV signature mutations, all trinucleotide classes that contain C>T mutations in Dipyr sequences (red circles with black outline in Figure 2C) show significant transcriptional asymmetry in WT cells, with ~2- to 3-fold higher mutation density on the NTS. Transcriptional asymmetry of these C>T mutations is elevated in the *rad16*Δ mutant (>10-fold asymmetry) and diminished in the *rad26*Δ mutant (Figure 2C), consistent with prior reports that these mutations arise from CPDs or 6–4PPs that are repaired by both NER sub-pathways. Similarly, certain classes of C>A mutations (Figure 2C; primarily TCN trinucleotide classes) and non-canonical UV-induced T>C mutations (Figure 2D) also show transcriptional asymmetry in WT cells that is further elevated in the *rad16*Δ mutant (Figures 2C–2E), indicating that these mutations also likely arise from bulky UV photoproducts. T>A mutations, however, appear to be caused by two separate UV-induced lesions. Lower abundance T>A mutations associated with TTC, TTG, and TTT sequences show transcriptional asymmetry favoring the NTS relative to the TS (Figures 2D and 2F), similar to T>C substitutions in these sequence contexts. Therefore, these mutations could originate from a TLS polymerase inserting a T at a lower frequency than a G across from the same bulky UV lesion that causes T>C mutations (likely a CPD or 6–4PP). In contrast, high-abundance T>A mutations in TTA trinucleotide sequences display transcriptional asymmetry favoring the TS (Figures 2D and 2F). Moreover, T>A mutations in contexts ending with a TA sequence (e.g., ATA, CTA, GTA, TTA) are all elevated on the TS relative to the NTS (Figures 2D and 2F). This analysis indicates that collectively “NTA” mutations originate from a DNA lesion on the opposite DNA strand, at a corresponding TAN consensus sequence (Figure 2G), and that the central adenine in this consensus is mutated to thymine (i.e., A>T mutation). T>A substitutions in an NTA sequence context total 1,677 mutations in WT cells, comprising 66% of T>A mutations and 12% of all mutations in our WT dataset, and therefore are a frequent UV-induced mutation.

### UV-Induced T>A Substitutions May Arise from TA Photoproducts

**Figure 3.**
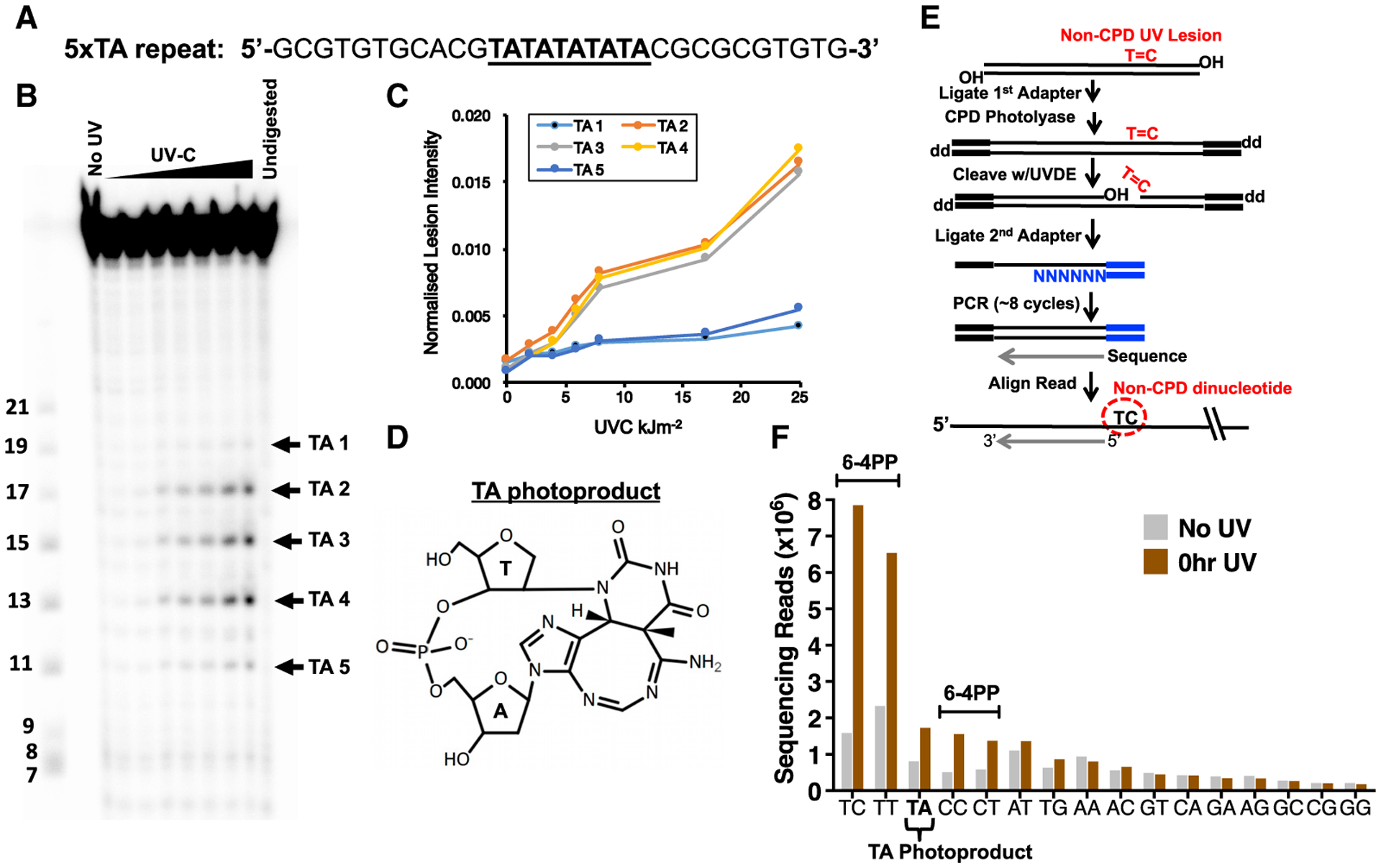
Mapping Atypical TA Photoproducts in UV-Irradiated DNA and Cells (A) Design of DNA oligonucleotide containing a stretch of five thymine-adenine (TA) sequences (underlined), but no dipyrimidine sequences. (B) Analysis of UV lesions by denaturing gel electrophoresis. Double-stranded DNA oligos were irradiated with increasing doses of UVC light (0.86–25.7 kJ/m^2^) and cleaved by UV DNA endonuclease (UVDE). The locations of the different TA photoproducts in the DNA sequence are indicated (TA 1–5), based on size standards in the leftmost lane. (C) Quantification of UVDE-cleaved TA photoproducts induced by different doses of UVC light. (D) Chemical structure of TA photoproduct. (E) UVDE-seq method for mapping non-CPD UV photoproducts. CPD lesions are removed by photoreactivation with purified CPD photolyase, and the remaining UV photoproducts are cleaved with UVDE. (F) UVDE-seq reads are enriched at dipyrimidine sequences and TA dinucleotides immediately following irradiation of a *rad16*Δ mutant with 600 J/m^2^ UVC light, consistent with UV-induced formation of 6–4PPs and TA photoproducts. “No UV” sample is included as a control.

Previous biochemical studies have indicated that UV light can induce a rare atypical photoproduct *in vitro* at TA dinucleotides (Bose et al., 1983; Zhao et al., 1996), which in theory could be responsible for inducing T-to-A mutations at NTA sequences. To characterize the formation of TA photoproducts, we UV-irradiated a double-stranded DNA oligonucleotide containing multiple TA sequences *in vitro* (Figure 3A). The UV-irradiated DNA was subsequently treated with UV DNA endonuclease (UVDE) from *Thermus thermophilus*, which cleaves a wide spectrum of UV photoproducts (Paspaleva et al., 2007), and the resulting products were separated by denaturing gel electrophoresis. We robustly detected products due to UVDE treatment with sizes consistent with cleavage at the TA sequences in a UV dose-dependent manner (Figures 3B and 3C), indicating UV exposure induced the formation of TA photoproducts (Figure 3D).

To examine whether UV induces TA photoproducts in cellular DNA, we mapped non-CPD DNA lesions at single-nucleotide resolution across the yeast genome using a new method called UVDE sequencing (UVDE-seq). This method is based on our previously published CPD-seq method (Mao et al., 2016), except CPD lesions are removed by CPD photolyase treatment *in vitro*, and the remaining UV damage is subsequently cleaved using UVDE (Figure 3E). UVDE-seq was used to map UV damage in repair-deficient yeast (i.e., *rad16*Δ) immediately following treatment with 600 J/m^2^ of UVC light (Figure 3F, “0 hr UV”) or in unirradiated yeast (Figure 3F, “No UV”). UVDE-seq reads were enriched at Dipyr sequences (Figure 3F), with the highest levels at TC sequences, followed by TT, CC, and CT. These abundances reflect the expected dinucleotide preferences for UVDE cutting at 6–4PPs. However, UVDE-seq reads at TA dinucleotides are also significantly enriched relative to the “No UV” control and represent the third most abundant non-CPD lesion (Figure 3F). Similar results were obtained using UVDE-seq to map UV photoproducts in WT cells (Figure S4), consistent with a previous report (Bryan et al., 2014). These data indicate that UV irradiation induces significant levels of TA photoproducts *in vitro* and across the yeast genome, which provides a plausible mechanism for the generation of UV mutations at TA sequences.

### Novel Tandem Mutations Associated with Atypical UV Photoproducts

**Figure 4.**
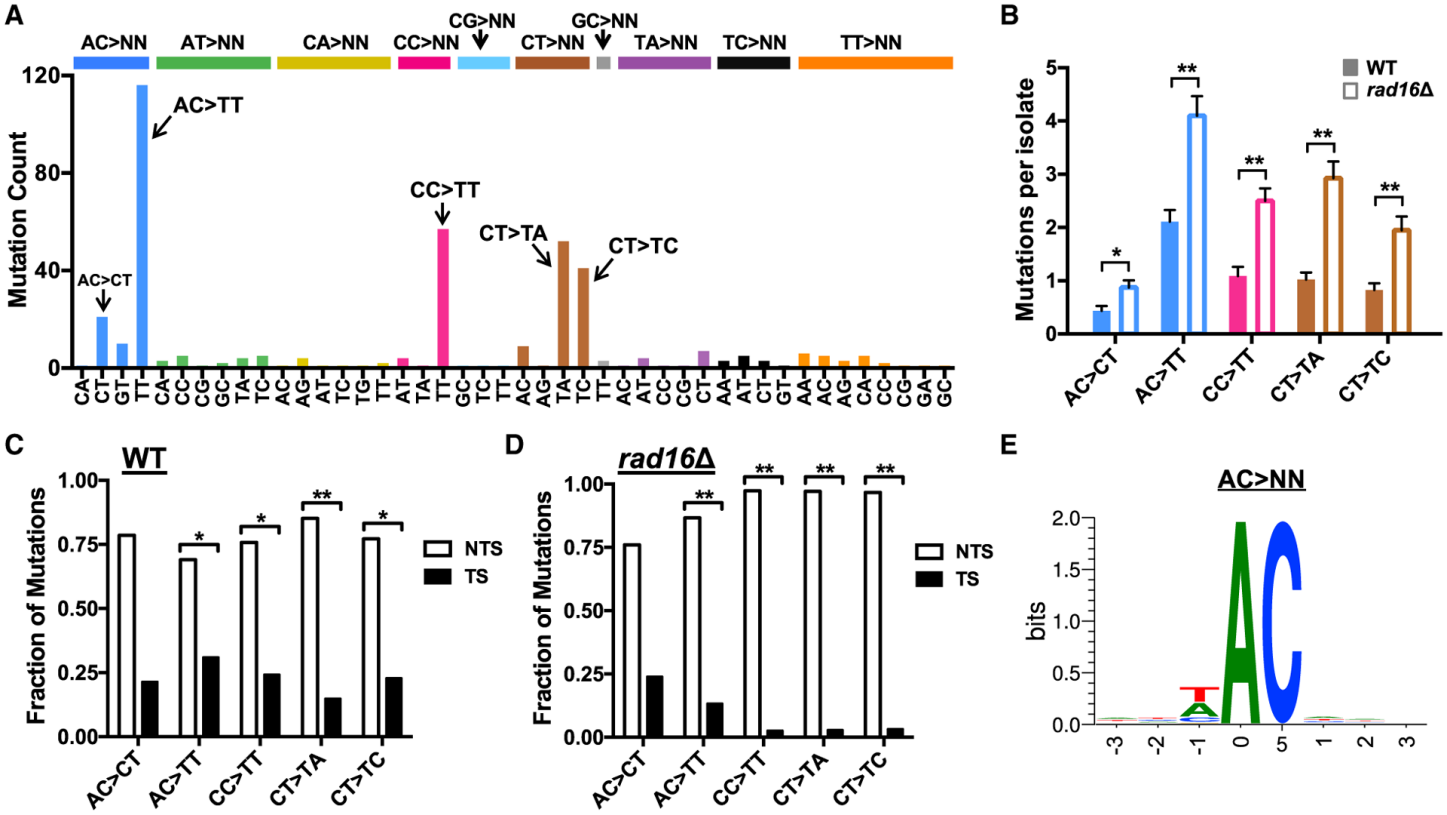
UV Light Induces Novel Tandem Mutations in Yeast (A) Spectrum of tandem mutations derived from genome sequencing of WT yeast following repeated UV exposure (9 or 15 doses). (B) UV-induced tandem substitutions are elevated in repair-deficient *rad16*Δ mutant cells. Mutations per sequenced isolate for 15 dose experiments are plotted. Significant differences in the number of mutations in each mutation class per isolate in WT relative to the *rad16*Δ mutant strain was determined using a t test with the Holm-Sidak correction for multiple hypothesis testing. **p < 0.001; *p < 0.01. (C and D) UV-induced tandem substitutions are elevated on the NTS of yeast genes in WT and *rad16*Δ mutant cells. Statistical significance was determined using the chi-square test and Bonferroni correction for multiple hypothesis testing. **p < 0.001; *p < 0.05. (E) Sequence logo representation of DNA flanking all AC>NN tandem substitutions (e.g., AC>TT, AC>CT, etc.) in yeast. Logo was generated using weblogo (Crooks et al., 2004).

UV irradiation also induced many tandem double substitutions (399 in WT) in yeast, but surprisingly, canonical UV-induced CC>TT mutations are only the second most frequent tandem mutation in this dataset (Figure 4A). The most frequent tandem mutations are instead AC>TT double substitutions, which are 2-fold more abundant than CC>TT mutations. CT>TA, CT>TC, and AC>CT tandem mutations are also common. Similar to UV-induced single-base substitutions, the frequency of each of these tandem mutations is elevated in *rad16*Δ mutant cells relative to WT (p < 0.05; Figure 4B). Most of these novel tandem mutations also show significant transcriptional asymmetry favoring the NTS (Figure 4C), which is exacerbated in *rad16*Δ mutant yeast (Figure 4D). These results indicate that along with the well-established CC>TT mutations, novel tandem mutations at AC and CT sequences may originate from UV lesions that are repaired by NER.

Although CT>NN tandem mutations likely arise because of mutagenic bypass of CPD or 6–4PPs forming at this Dipyr sequence, UV photoproducts have not been previously identified at adenine-cytosine dinucleotides. Analysis of all AC>NN mutations in yeast revealed little, if any, sequence conservation in flanking DNA (Figure 4E). Indeed, 50 of the AC>NN mutations occur in a TACA sequence context, which has no overlapping Dipyr sequences. Therefore, AC>NN mutations are unlikely to be caused by mutagenic bypass of canonical UV photoproducts at neighboring or overlapping Dipyr sequences and, similar to A>T mutations in NTA sequences, are likely to be caused by an atypical UV photoproduct.

### Skin Cancer Genomes Contain Non-canonical T>C, A>T, and AC>NN Mutations

**Figure 5.**
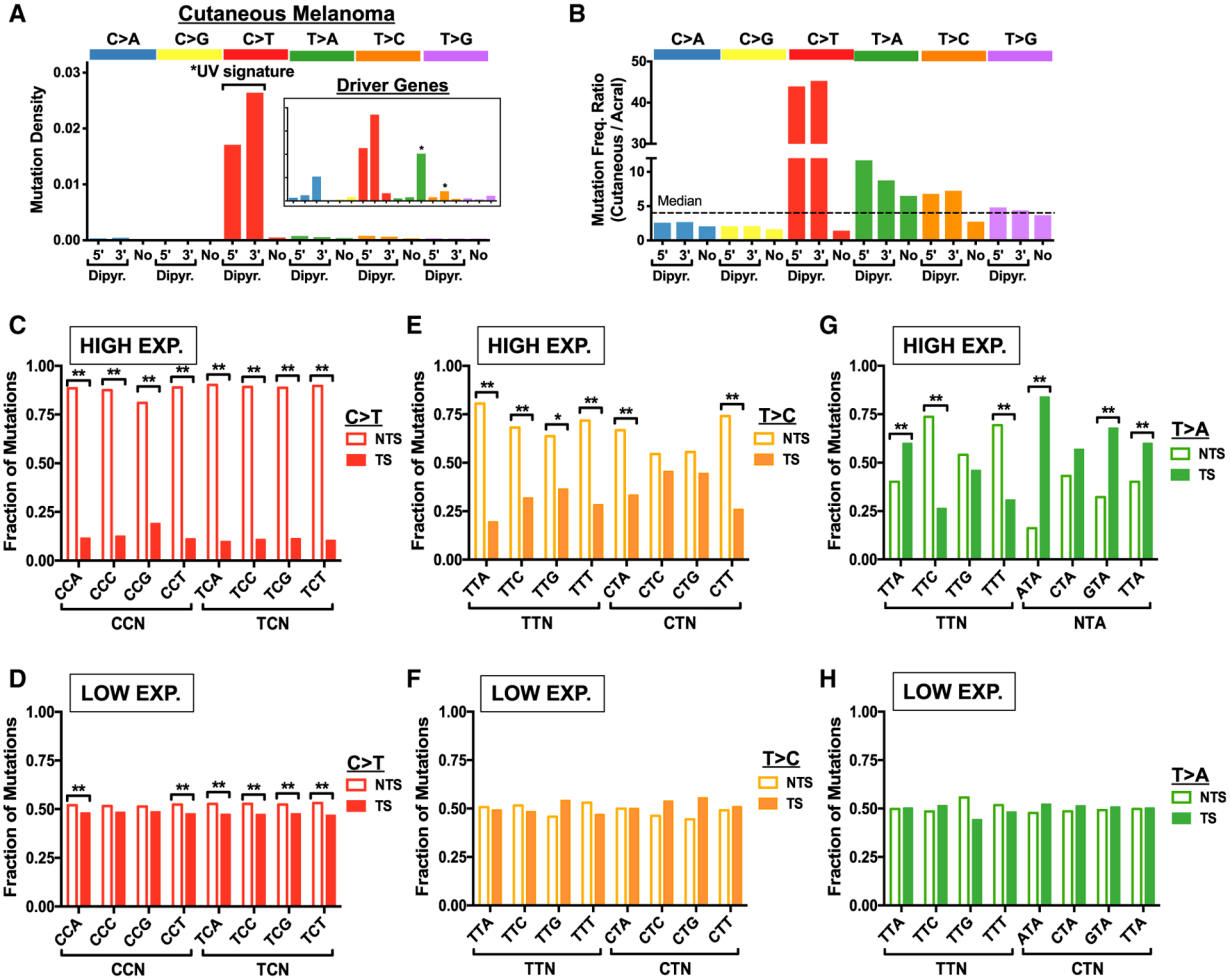
Non-canonical Mutation Classes in Skin Cancers Are Associated with UV Exposure and Show Transcriptional Asymmetry (A) Density of mutations for 140 sequenced cutaneous melanomas (Hayward et al., 2017) associated with the 5′ position of dipyrimidine (Dipyr.), 3′ position of dipyrimidine, or not associated with a dipyrimidine (No), as defined in Figure S1C. Inset shows data just for mutations in coding exons of melanoma driver genes. (B) Ratio of mutation frequency per tumor for 140 cutaneous melanomas relative to 35 acral melanomas (Hayward et al., 2017). Dashed line indicates the median ratio across all mutation classes. (C and D) Fraction of C>T mutations in genes occurring on the NTS relative to the TS for the indicated trinucleotide contexts in cutaneous squamous cell carcinomas (cSCCs) derived from *XPC*^−/−^ patients is plotted. Transcriptional asymmetry is plotted for genes (C) highly expressed (top quartile) and (D) lowly expressed (bottom quartile) in keratinocytes. Statistical significance was determined using the chi-square test and Bonferroni correction for multiple hypothesis testing. **p < 0.001; *p < 0.05. (E and F) Same as (C) and (D), except for T>C mutations. (G and H) T>A mutations are significantly enriched on the TS relative to the NTS at NTA sequences (e.g., ATA, GTA, TTA) in *XPC*^−/−^ cSCCs in genes highly expressed (top quartile; G), but not in low-expressed genes (bottom quartile; H).

Given the striking abundance of non-canonical UV-induced mutations in our yeast dataset, we next assessed whether similar types of mutations are present in the genomes of human cancers associated with UV exposure. Initially, we analyzed single-nucleotide substitutions derived from whole-genome sequencing of 140 cutaneous melanoma tumors (Hayward et al., 2017) in a manner similar to our yeast data. This effort revealed T>C and T>A substitutions comprise only 5% and 4% of total single-nucleotide substitutions, respectively, because the vast majority of substitutions in these tumors are UV signature mutations (Figure 5A). Despite their lower abundance compared with our yeast dataset, T>C and T>A mutations are enriched in cutaneous melanomas relative to acral melanomas (which are not typically UV exposed). T>C and T>A mutation classes are elevated ~6- to 12-fold in cutaneous relative to acral melanoma (Figure 5B). Although this enrichment is not as high as UV signature C>T mutations (>40-fold enrichment), it is higher than other mutation classes (Figure 5B), indicating at least a subset of T>C and T>A mutations may be UV induced.

Abundances of T>C and T>A mutations similar to those in cutaneous melanoma were also observed in a group of sun-exposed cSCCs (Figures S5A and S5B), which, although they differ from melanoma in terms of causative driver mutations, share an association with UV exposure. Importantly, this dataset also contains sequenced cSCCs derived from patients with germline mutations in *XPC* (Zheng et al., 2014), which like Rad16 in yeast, is required for GG-NER in human cells. We therefore compared the frequency of different mutations classes in WT and *XPC*^−/−^ cSCCs. The frequency of UV signature C>T mutations is elevated in NER-deficient *XPC*^−/−^ cSCCs relative to repair-proficient cSCCs (Figure S5C), as expected. The mutation densities for a number of T>C and T>A mutation classes are also elevated in the NER-deficient *XPC*^−/−^ cSCCs (Figures S5D and S5E), consistent with many of these mutations being induced by bulky DNA lesions. In particular, T>A mutations in an NTA sequence context (i.e., ATA, CTA, GTA, and TTA) are highly elevated in *XPC*^−/−^ cSCCs (Figure S5E).

Analysis of canonical UV signature C>T mutations revealed significant transcriptional asymmetry in genes that are highly expressed in keratinocytes (top quartile) in the *XPC*^−/−^ cSCCs (Figure 5C). In contrast, transcriptional asymmetry is much lower in low-expressed genes (bottom quartile; Figure 5D), likely due to low TC-NER activity in the absence of ongoing transcription. Similarly, non-canonical UV-induced T>C mutations revealed a significant transcriptional asymmetry for most Dipyr mutation classes among high-expressed genes (Figure 5E), but not in low-expressed genes (Figure 5F), although the magnitude of transcriptional asymmetry for T>C mutations is somewhat lower than C>T mutations. As in yeast, T>A mutations in an “NTA” sequence context are elevated on the TS in highly expressed genes (Figure 5G), but not in lowly expressed genes (Figure 5H), suggesting that many of the T>A mutations may arise from atypical TA photoproducts (Figure 2G). Similar trends in transcriptional asymmetry are apparent for T>C and T>A mutations in cutaneous melanoma (Figures S6A–S6D), although the magnitude of transcriptional asymmetry is decreased, presumably because of active GG-NER in these tumors. These findings are consistent with the hypothesis that many T>C and T>A mutations in cutaneous melanoma and cSCC are induced by UV exposure.

**Figure 6.**
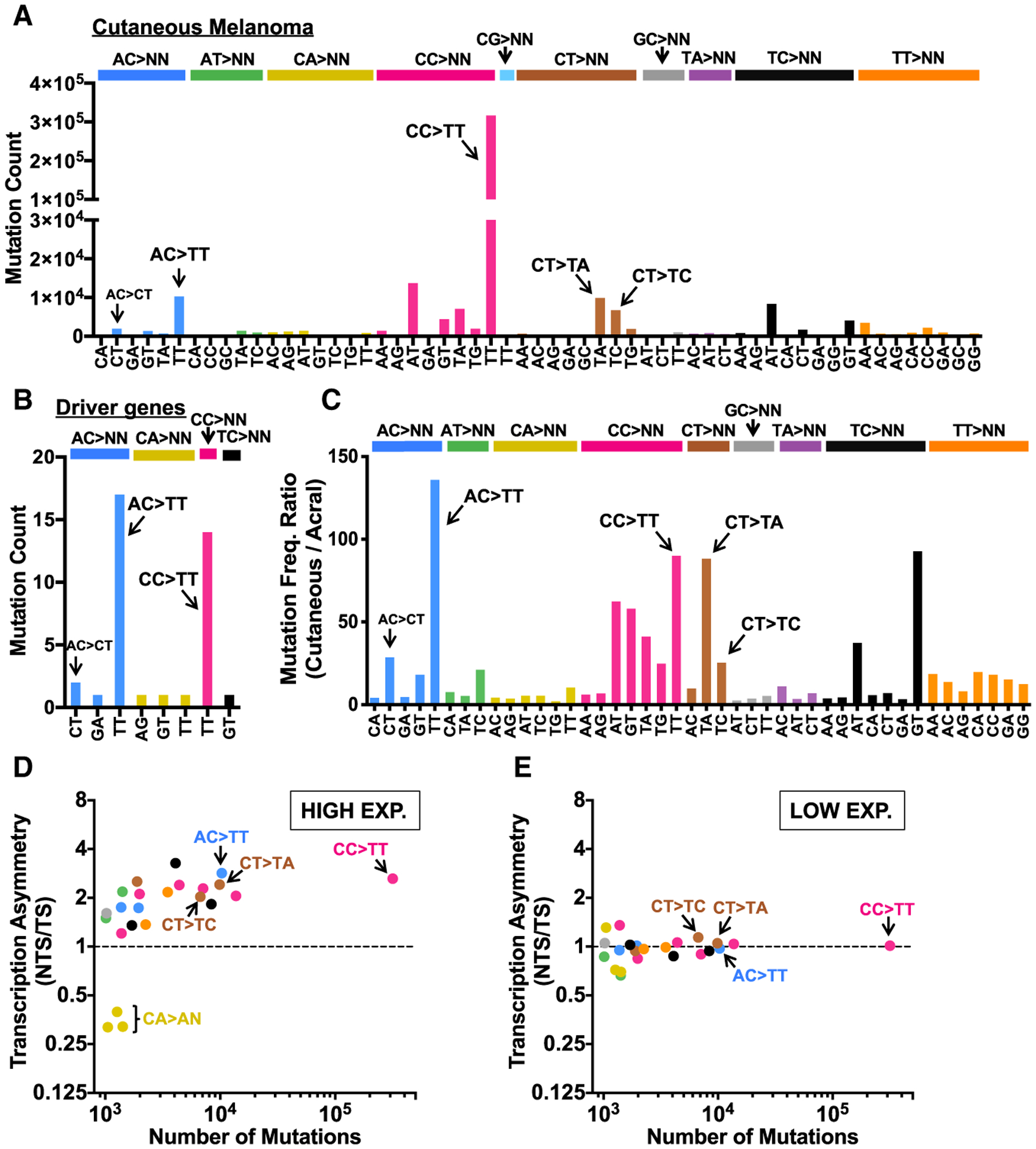
Non-canonical Tandem Mutations in Melanoma Are Associated with UV Exposure and Show Transcriptional Asymmetry (A) Spectrum of tandem mutations in 140 sequenced cutaneous melanomas (Hayward et al., 2017). (B) Spectrum of tandem mutations in the coding exons of melanoma driver genes. (C) Ratio of tandem mutation frequency per tumor for 140 cutaneous melanomas relative to 35 acral melanomas (Hayward et al., 2017). Only tandem mutation classes with at least 150 mutations in the cutaneous melanoma dataset and 10 mutations in the acral melanoma dataset are plotted. (D) Transcriptional asymmetry (normalized ratio of NTS relative to TS) of tandem mutations in high-expressed melanocyte genes (top quartile) plotted versus total number of each type of tandem mutation. (E) Same as (D), except transcriptional asymmetry is plotted for tandem mutations in low-expressed melanocyte genes (bottom quartile).

To investigate whether AC>NN and other novel tandem mutation classes observed in yeast occur in skin cancers, we analyzed tandem mutations in the cutaneous melanoma dataset. Although CC>TT tandem mutations are by far the most common double substitution in cutaneous melanoma, as expected, significant numbers of AC>TT, CT>TA, and CT>TC tandem mutations are also present (Figure 6A). Among melanoma driver genes, however, AC>TT is the most common tandem mutation (Figure 6B). Many of these classes of tandem mutations are elevated in cutaneous relative to acral melanomas (Figure 6C). For example, UV signature CC>TT mutations are 90-fold more abundant in cutaneous relative to acral melanoma. Similar or even higher levels of enrichment were observed for AC>TT and CT>TA tandem substitutions (Figure 6C). UV signature CC>TT mutations are also elevated ~2.6-fold on the NTS relative to TS in highly expressed genes (Figure 6D), but show no transcriptional asymmetry in low-expressed genes (Figure 6E). A similar degree of transcriptional asymmetry is observed for AC>TT, CT>TA, CT>TC, and other non-canonical tandem mutations (Figures 6D and 6E), indicating these mutation classes may also originate from UV photoproducts repaired by TC-NER. Notably, CA>AN tandem mutations show the reverse transcriptional asymmetry (Figure 6D), indicating that the originating DNA lesion occurs at a TG sequence. CA>NN mutations are rare in our yeast dataset and show relatively little enrichment in cutaneous relative to acral melanomas (Figure 6C), so it is unclear whether they are directly induced by UV exposure. Finally, like AC>NN mutations in yeast, there is relatively little sequence specificity flanking AC>NN mutations in cutaneous melanoma (Figure S6E), indicating these mutations are unlikely to be a consequence of UV photoproducts forming at neighboring or overlapping Dipyr sequences.

### Novel UV-Induced Mutations Can Explain Recurrent Mutations in Melanoma

**Figure 7.**
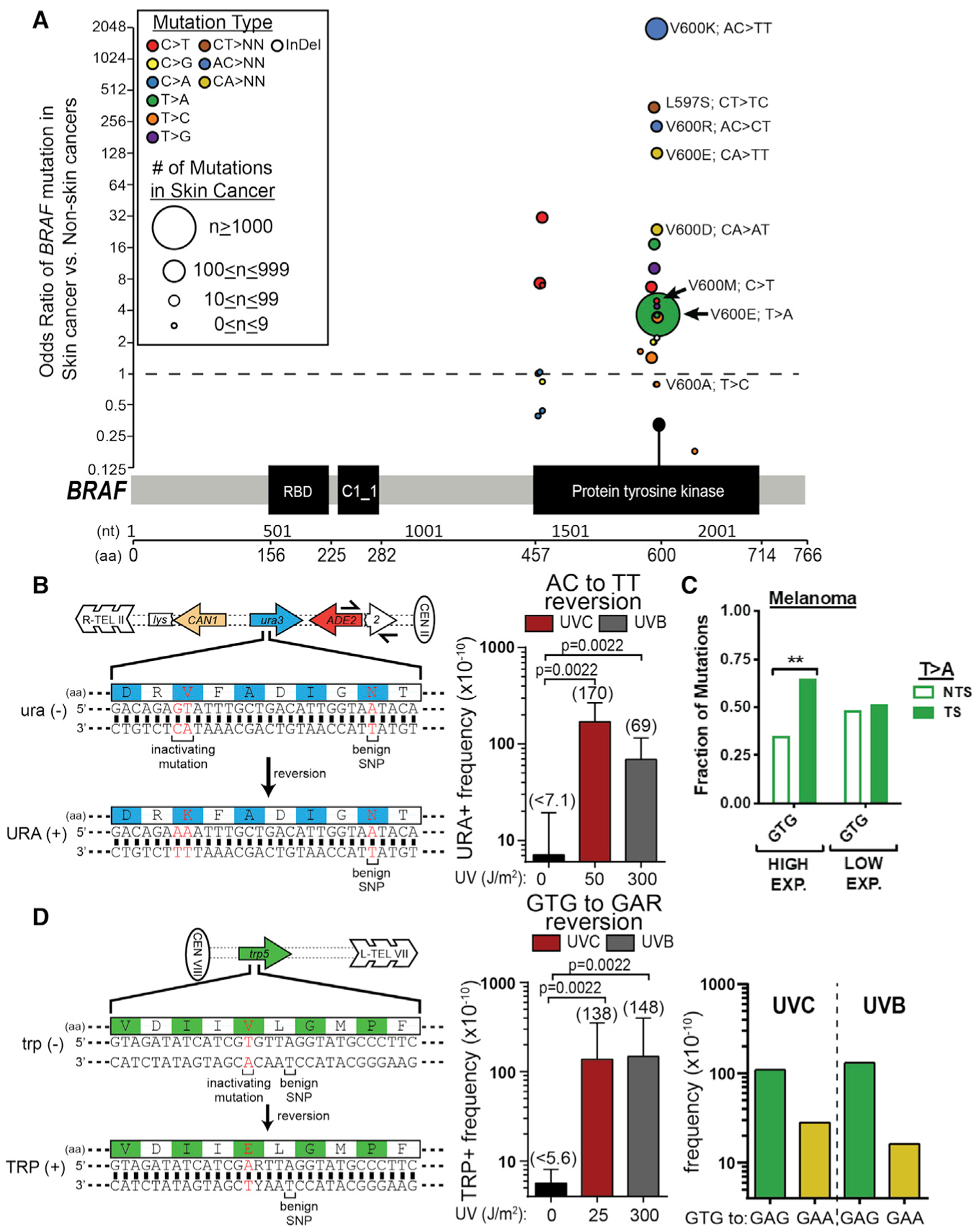
UV Light Induces Oncogenic Tandem Substitutions Found in BRAF (A) Odds ratio of recurrent substitution mutations (circles) in the *BRAF* gene in skin cancers relative to non-skin cancers from the COSMIC database (Tate et al., 2019). Each mutation is positioned along the x axis in accordance with its position within the BRAF cDNA. Nucleotide (nt) and amino acid (aa) positions are indicated below the schematic of the *BRAF* protein domains. Specific substitution types are color coded, and the number of times each recurrent mutation occurs in the dataset is indicated by the size of the circle. (B) UV light induces AC-to-TT tandem substitutions in yeast. The yeast *ura3* K93V reporter is inactive due to an AA-to-GT (red text) substitution at codon 93, resulting in the change of the catalytic lysine (Miller et al., 2001) to valine. Reversion of the *ura3* K93V to WT via an AC>TT mutation therefore mimics the *BRAF* V600K substitution. Median URA^+^ reversion frequencies were determined from six independent measurements for yeast treated with 0, 50 J/m^2^ UVC light, or 300 J/m^2^ UVB light. Error bars indicate ranges. For yeast treated with 0 J/m^2^ UVC, no URA^+^ revertants were recovered in any of the six replicates, so a maximum estimated frequency was calculated as if each replicate contained a single URA^+^ colony. p = 0.0022 by Mann-Whitney ranked sum test. (C) T>A mutations in a GTG context are significantly enriched on the TS relative to the NTS in highly expressed genes in melanocytes (top quartile), but not in lowly expressed genes (bottom quartile). **p < 0.001. (D) UV light induces T>A and TG>AA substitutions in a GTG context. Same as (B), except the yeast *trp5* E50V reversion reporter was used to mimic the *BRAF* V600E mutation. Median TRP^+^ reversion frequencies were determined from six independent measurements for yeast treated with 0, 25 J/m^2^ UVC light, or 300 J/m^2^ UVB light. Error bars indicate ranges. p = 0.0022 by Mann-Whitney ranked sum test. Right panel: estimated frequency of T>A (i.e., GTG-to-GAG) and TG>AA (i.e., GTG-to-GAA) mutations in the *trp5* reversion assay, based on sequencing of TRP^+^ revertants.

Oncogenic *BRAF* and *NRAS* mutations are the most common melanoma driver mutations, yet these mutations typically do not match the UV signature. We wondered whether the non-canonical UV-induced mutations identified in this study could explain the occurrence of some *BRAF* and *NRAS* mutations, thereby providing a functional link between UV exposure and melanomagenesis. We obtained the mutation status of the *BRAF* gene in 304,517 samples available from the Catalog of Somatic Mutations in Cancer (COSMIC) database (Tate et al., 2019) and accessed the odds ratio for specific mutations to occur in skin cancers as compared with other cancer types. Because skin cancers are the predominant cancer type whose etiology is associated with exposure to UV irradiation, mutations for which UV-induced damage was a key underlying cause would be expected to be highly enriched within skin cancer. As expected, the *BRAF* mutations within our dataset primarily cluster at the known oncogenic V600 position (Figure 7A). Of the BRAF V600 variants, the minor variant V600M is the only UV signature mutation (i.e., C>T substitution in a Dipyr context). V600M is 4.9-fold more likely to occur in skin cancers as opposed to non-skin cancers, consistent with its potential induction by UV exposure. Although the remaining *BRAF* V600 variants are non-UV signature mutations, many of these are highly enriched in skin cancers. For example, *BRAF* V600K mutations occur almost exclusively in skin cancers, displaying a nearly 2,000-fold greater likelihood of being observed in UV-exposed tissue as compared with other tumor types (Figure 7A). The *BRAF* V600K mutation is the second most common *BRAF* variant in the analyzed dataset (occurring in 537 samples) and is an AC>TT (or GT>AA) tandem substitution. Similarly, the *BRAF* V600R mutation is highly enriched in skin cancers (Figure 7A) and is caused by an AC>CT (or GT>AG) tandem substitution. Our analysis of AC>CT and AC>TT mutations in yeast and human cells indicate that these driver mutations are likely induced by an atypical UV photoproduct occurring at the AC dinucleotide on the TS of the *BRAF* gene. Among non-V600 variants, the *BRAF* L597S, which is due to a non-UV signature CT>TC tandem substitution, is also highly enriched in skin cancers. Tandem oncogenic mutations in *BRAF* are also enriched in skin cancers relative to thyroid cancer, which also commonly involves oncogenic *BRAF* mutations (Table S1).

Similarly to recurrent *BRAF* mutations, non-canonical C>T, C>A, T>A, and T>C mutations in *NRAS* are 2.6- to 38-fold enriched within skin cancers compared with non-skin cancers (Figure S7). *NRAS* Q61R and Q61L involve T>C and T>A substitutions, respectively, within a TTG context, which are consistent with experimentally derived UV-induced mutations we observed in yeast (Figure 2).

Our data suggest that *BRAF* V600K mutations arise from a single AC>TT tandem substitution event; however, it is theoretically possible that these mutations arise from two independent mutational events, perhaps occurring in separate UV exposures. In addition, it is unclear whether lower-frequency UVB light, which is the primary exposure risk in sunlight, can induce an AC>TT substitution. To test whether a single dose of either type of UV light can induce the tandem substitution required to cause oncogenic *BRAF* V600K mutations, we engineered a yeast strain to contain an inactive *ura3* allele, in which the catalytic K93 residue of Ura3 is mutated to valine by an AA-to-GT change in the coding strand (Figure 7B). Reversion of this mutant Ura3 to an active form would therefore require a V93K mutation and mimic the mutational process required to generate a *BRAF* V600K mutation. Treatment of yeast containing this *ura3* reversion reporter with a single dose of either 50 J/m^2^ UVC light or 300 J/m^2^ UVB light induces URA^+^ cells at a low, but consistently detectable frequency (i.e., 4–24 URA^+^ colonies per ~1 billion cells). The calculated reversion frequency for UVC light (i.e., 130 × 10^−10^; see Figure 7B) is roughly consistent with the expected value of ~700 × 10^−10^, based on the frequency of AC>TT tandem mutations in the sequenced WT isolates. In contrast, no URA^+^ revertants were observed in the absence of UV exposure (~10 billion cells plated in total). Therefore, we calculated the reversion frequency in this condition is a maximum estimate, as if one URA^+^ isolate were obtained per replicate experiment (Figure 7B). Sequencing of URA^+^ isolates confirmed that all URA^+^ isolates obtained following UVC and UVB exposure are true reversion mutations because they contained the expected GT>AA mutation needed to restore Ura3 function, while maintaining a benign SNP engineered into the reversion strain during its construction.

The *BRAF* V600E mutation is the most frequent driver mutation in melanoma. Despite this mutation primarily involving a T-to-A substitution in a GTG sequence context, the V600E mutation was 3.6-fold more frequent in skin cancers than in non-skin cancers (nearly as enriched as V600M), suggesting that some of these mutations may be induced by UV exposure in skin cancers. Analysis of sequenced cutaneous melanomas revealed that T>A substitutions in a GTG sequence context show significant transcriptional asymmetry in highly expressed genes (Figure 7C), with mutations being elevated on the TS. In contrast, there is no transcriptional asymmetry in low-expressed genes (Figure 7C). This analysis suggests that at least a subset of these mutations may arise from a UV-induced photoproduct occurring in the CAC sequence on the opposite strand. The *BRAF* V600E mutation is also occasionally caused by a CA>TT (i.e., TG>AA) tandem substitution, which, although infrequent, is highly enriched in skin cancers (~130-fold; Figure 7A).

To test the hypothesis that the *BRAF* V600E mutation is UV inducible, we adapted a published mutation reporter (Williams et al., 2005), in which the yeast *TRP5* gene is inactivated by an E50V mutation. We measured the frequency in which the mutant *trp5* gene in yeast is reverted to an active form (i.e., TRP^+^) by a V50E substitution. This reversion would require a T>A substitution in a GTG context, thereby modeling the *BRAF* V600E substitution (Figure 7D). Irradiation of the *trp5* reversion strain with a single dose of 25 J/m^2^ UVC or 300 J/m^2^ UVB light consistently induced TRP^+^ colonies (14–37 colonies per ~1–2 billion cells plated), with median reversion frequencies of 138 × 10^−10^ and 148 × 10^−10^, respectively (Figure 7D). In contrast, very few TRP^+^ colonies were obtained in the absence of UV (total of 3 TRP^+^ colonies from ~16 billion cells plated in total for 0 J/m^2^ UVC), so the 0 J/m^2^ reversion frequency was calculated as a maximum estimate, as if at least one TRP^+^ isolate were obtained per replicate experiment (Figure 7D). Sequencing of the UV-induced TRP^+^ colonies revealed a mix of T>A (80%, 39 out of 49 TRP^+^ colonies, for UVC; 89%, 41 out of 46 TRP^+^ colonies, for UVB) and TG>AA (i.e., CA>TT on TS; 20%, 10 out of 49 TRP^+^ colonies, for UVC; 11%, 5 out of 46 TRP^+^ colonies, for UVB) substitutions, indicating that UV light can induce both types of *BRAF* V600E mutations. The overall reversion frequency and the relative abundance of each mutation type were used to estimate the frequency of UV-induced T>A and TG>AA (i.e., CA>TT) substitutions (Figure 7D, right panel). The three TRP^+^ colonies isolated following no UV irradiation (0 J/m^2^ UVC) were all T>A substitutions.

## DISCUSSION

Our results reveal that UV light can induce a more diverse spectrum of mutations than previously suspected. Genome sequencing of >150 independent yeast isolates repeatedly irradiated with UVC light revealed four novel mutation classes (Table S2). These mutation classes comprise nearly 50% of the ~50,000 mutations identified in our study, highlighting their potential significance to UV mutagenesis. Our analysis further suggests that similar mutation classes may be present in human skin cancers, albeit at lower frequencies. Genome-wide mutation and lesion-mapping data suggest that two of these mutation classes are caused by atypical UV photoproducts, namely, a TA photoproduct and an unknown UV-induced lesion at adenine-cytosine dinucleotides. Surprisingly, non-canonical mutation signatures detected in UV-irradiated yeast provide a potential mechanism for some of the most frequent oncogenic mutations in melanoma (Table S2). Our work suggests that UV exposure may stimulate oncogenic mutations in *BRAF* and potentially other melanoma driver genes by inducing the formation of rare, but highly mutagenic, photoproducts.

In contrast with the results reported here, prior studies of UV mutagenesis in mammalian cells have concluded that short- or medium-wavelength UV light (i.e., UVC and UVB) specifically induce C>T and CC>TT substitutions (Brash, 2015; Ikehata and Ono, 2011; Pfeifer et al., 2005). For example, a recent genome-wide survey of mutagenesis in human cells reported that only UV signature mutations (i.e., C>T and CC>TT) were enriched following a single low dose of UV (Kucab et al., 2019). This survey also identified other mutations in UV-irradiated cells, including many of the same mutation classes identified in our study (i.e., T>A and T>C), but their lower abundance, coupled with the occurrence of high numbers of background mutations in all cell lines, resulted in these mutation classes being overlooked. Similarly, non-UV signature mutations are abundant in skin cancers, but these mutation classes have been overshadowed by the predominance of UV signature mutations in these tumors.

The sensitivity of our approach in detecting and characterizing novel UV mutation signatures can be attributed to multiple aspects of the experimental design. First, diploid yeasts are much more tolerant of UV light than mammalian cells, so yeast can be repeatedly exposed to relatively high UV doses and thereby accumulate mutations arising from potentially rare UV photoproducts. Second, the rapid proliferation of yeast cells ensures that even rare or rapidly repaired DNA photoproducts persist to S-phase and therefore contribute to mutagenesis. For example, it is likely that many of the T>C mutations detected in our study arise from 6–4PPs (Bresson and Fuchs, 2002), yet these UV photoproducts are so rapidly repaired in mammalian cells (Adar et al., 2016) that few persist through the longer mammalian cell cycle. Third, yeast have a much lower number of background mutations than mammalian cells grown in cell culture, ensuring that the vast majority of mutations in yeast arise as a direct consequence of UV exposure. Fourth, measuring UV mutagenesis in repair-deficient yeast strains (e.g., *rad16*Δ and *rad26*Δ) is a robust method for determining which classes of mutation are caused by bulky UV photoproducts, as well as identifying the DNA strand in which the lesion occurred (e.g., AC versus GT). Finally, the large number of independent clonal isolates (>150) that were sequenced in our study provides the power to detect and characterize potentially rare UV mutation classes. In contrast, only three UV-exposed mammalian cell clones were sequenced in a previous genome survey (Kucab et al., 2019), presumably because of the increased time and expense required to sequence the much larger human genome. These inherent advantages of the yeast model system could be exploited to identify non-canonical or rare mutation signatures associated with other mutagens.

An additional feature of our study was the use of UVC light to induce mutations. Although UVC light induces a similar spectrum of DNA lesions as solar UVB light, it more strongly induces less common or rare UV lesions, such as 6–4PPs (Besaratinia et al., 2011; Friedberg et al., 2006). This property of UVC light likely enabled us to more readily identify non-canonical UV mutation signatures by enriching for such rare photoproducts. Importantly, we show that a number of these non-canonical mutations can also be induced by UVB exposure (Figure 7). Recent studies have also found T>C mutations and potentially other non-canonical UV mutations in UVB-exposed *C. elegans* (Volkova et al., 2020) and sun-exposed normal human skin (Saini et al., 2016), consistent with our findings. However, many of the T>C mutations in human cells are associated with NTT sequences (i.e., the 5′ position of a Dipyr sequence is mutated, not the 3′ position), and therefore may represent a distinct mutational signature, possibly because of differences in lesion bypass by translesion DNA polymerases.

The most unusual class of mutation discovered in this study is the AC>CT and AC>TT double substitutions, the latter of which occurs more frequently than CC>TT tandem mutations in UVC-exposed yeast. Given their frequency, it is remarkable that this class of UV mutations has not previously been reported or analyzed in any systematic way. There have been hints in the literature that UV light can cause AC>TT mutations (e.g., Reis et al., 2000), perhaps the best-known example being the UV-induced lacUV5 promoter mutation (Silverstone et al., 1970). This mutation class is highly relevant to melanoma, because these tandem substitutions are responsible for the oncogenic *BRAF* V600R and V600K mutations. Our analysis indicates that the *BRAF* V600R and V600K mutations occur almost exclusively in skin cancers, consistent with their UV origin. Moreover, epidemiological data indicate that the *BRAF* V600K mutations are associated with melanomas occurring in chronic sun-exposed areas of the head and neck, and are more prevalent in geographical regions with higher ambient UV light (Kong et al., 2016). It has been previously suggested that these tandem *BRAF* mutations arise from mutagenic bypass of canonical UV lesions occurring at neighboring/overlapping Dipyr (Thomas et al., 2006). However, our genome-wide analysis of AC>NN mutations in yeast and human cells indicates there is little, if any, sequence bias in neighboring nucleotides and therefore is inconsistent with this model. Moreover, AC>TT mutations occur frequently in sequence contexts that lack any neighboring Dipyr sequences. The simplest explanation for our data is that these tandem mutations originate from an atypical UV photoproduct forming at AC sequences. That such a photoproduct has been overlooked is not surprising, particularly if it is highly mutagenic and occurs at low abundance.

Our data indicate that atypical UV photoproducts may be responsible for other novel mutation signatures in UV-exposed yeast and human skin cancers. For example, multiple lines of evidence indicate that UV-induced A>T substitutions in yeast are primarily due to non-canonical TA photoproducts. Perhaps the most convincing line of evidence is that UV-induced A>T mutations in TA sequences have significant strand asymmetry in transcribed genes, both in yeast and human cells, which is modulated by the NER pathway. This analysis highlights the power of transcriptional asymmetry to elucidate novel mutational processes or mutagenic lesions, particularly when applied to mutation data from repair-deficient cells (e.g., *rad16*Δ or *XPC*^−/−^) or high- versus low-expressed genes. TA photoproducts were discovered decades ago (Bose et al., 1983), but their potential contribution to UV mutagenesis was previously unclear. Our data indicate that TA photoproducts are highly mutagenic *in vivo* due to misinsertion of an adenine nucleotide opposite the 3′ adenine base in the TA photoproduct. This signature matches a reported mutation spectra of a site-specific TA photoproduct when transformed into *E. coli* (Zhao and Taylor, 1996) and can be potentially explained by structural studies (Davies et al., 2007; Zhao et al., 1996; Zhao and Taylor, 1996).

There are telltale signs that other atypical photoproducts may contribute to UV mutagenesis. Of particular interest are T>A mutations in a GTG context, which are responsible for the oncogenic *BRAF* V600E mutation. This is the most frequent driver mutation in melanoma, yet it is not thought to originate from UV damage because of its non-canonical substitution pattern and non-Dipyr sequence context. Using a yeast reversion reporter, we show that T>A mutations in a GTG context are UV inducible. Furthermore, transcriptional asymmetry analysis indicates that this mutation class may originate from an unknown bulky DNA lesion occurring in the CAC sequence on the opposite strand. One possibility is that the putative photoproduct at AC dinucleotides may cause not only AC>TT mutations, but also single A>T substitutions. Alternatively, a previously reported photoproduct at CA dinucleotides (Su et al., 2010) could induce these mutations. Consistent with this hypothesis, a significant fraction of the UV-induced TRP^+^ revertants in our yeast assay were CA>TT tandem substitutions, a mutation class that causes the complex BRAF V600E variant in melanoma (Thomas et al., 2004). The yeast reversion assays described in this study could be used to investigate whether other mutagens cause these recurrent *BRAF* mutations, essentially as a type of Ames test (Ames et al., 1973) for the causes of oncogenic mutations found in melanoma and other cancers.

The concept that rare atypical photoproducts may function as oncogenic lesions in melanoma represents a new paradigm that can potentially explain many of the unique aspects of melanoma epidemiology. For example, epidemiological studies have indicated that melanoma risk is associated with incidents of severe sunburn (Garibyan and Fisher, 2010). Our model suggests that the high UV dose associated with severe sunburn may be required to generate the rare atypical photoproducts needed to induce the *BRAF* (or *NRAS*) driver mutations important for melanomagenesis. Similarly, the late onset of melanoma in XP patients relative to non-melanoma skin cancers (DiGiovanna and Kraemer, 2012) could be explained by the requirement for these same rare atypical photoproducts. Our results ultimately suggest that in the case of melanoma, the real culprit may not be the multitude of canonical lesions (i.e., CPDs and 6–4PPs) formed during UV exposure, but rather the rare atypical photoproducts that have been concealed in their midst.

## STAR★METHODS

### RESOURCE AVAILABILITY

#### Lead Contact

All requests for further information, materials, yeast strains, resources and reagents should be directed to and will be fulfilled by the Lead Contact of the manuscript, John J. Wyrick (jwyrick@wsu.edu.).

#### Materials Availability

All yeast strains and other materials generated in this study (Key Resources Table) will be made available upon request to the Lead Contact and completion of an appropriate material transfer agreement.

#### Data and Code Availability

The UVDE-seq data has been submitted to the NCBI Gene Expression Omnibus (GEO; https://www.ncbi.nlm.nih.gov/geo/) under accession number GSE144679. Raw fastq files containing the sequencing data used to identify UV-induced mutations in whole genome sequenced yeast are available at NCBI Sequence Read Archive (SRA; https://www.ncbi.nlm.nih.gov/sra) under BioProject accession number PRJNA605561. UV-induced mutation calls in yeast are provided in Data S1. All code used to count mutation contexts, assess transcriptional asymmetry, and analyze UVDE-seq data are available from the lead contact upon request. Original data containing lists of melanoma mutations, cSCC mutations, and RNA-seq for melanocytes and keratinocytes can be found at https://dcc.icgc.org/api/v1/download?fn=/release_20/Projects/MELA-AU/simple_somatic_mutation.open.MELA-AU.tsv.gz, (Zheng et al., 2014), and https://egg2.wustl.edu/roadmap/data/byDataType/rna/expression/, respectively. *BRAF* and *NRAS* mutation status in human cancer samples are available from https://cancer.sanger.ac.uk/cosmic/gene/analysis?ln=BRAF and https://cancer.sanger.ac.uk/cosmic/gene/analysis?ln=NRAS.

### EXPERIMENTAL MODEL AND SUBJECT DETAILS

#### Yeast Stains

Yeast stains utilized in this study were constructed in either the BY4741, BY4742 or ySR128 genetic backgrounds. ySR128 was previously constructed in the CG379 yeast background, which is a derivative of S288C (Roberts et al., 2012). Yeast were grown either in YPD medium, on synthetic complete (SC) medium, SC lacking uracil (SC-Ura), SC lacking tryptophan (SC-Trp), or SC lacking arginine and supplemented with canavanine (SC-Arg+Can) at 30þC, depending on whether mutagenesis would be assessed by whole genome sequencing, reversion assay, or forward mutation assay. Yeast subjected to UVDE-seq were grown in YPD media at 30þC.

#### E. coli

Expression of UVDE and CPD photolyase for the purification of these enzymes was conducted in BL21 pLysS and T7 express cells, respectively. In both cases, the *E. coli* were grown in LB broth at either 30þC or 37 þC, as specified in the method details.

### METHOD DETAILS

#### Genetic Modification of Yeast Strains

Diploid strains were used for all UV passaging experiments. The wild-type BY4743 is a product of mating BY4741 and BY4742. The *rad26*Δ diploid strain was created by mating *rad26*Δ yeast in the BY4741 and BY4742 backgrounds (made by *TRP1* and *LEU2* insertion, respectively) with each other and confirming by selection on plates and PCR analysis. The *rad16*Δ diploid strain was created by mating knockout strains (again, using *TRP1* and *LEU2* selection) created in BY4741 and BY4742 backgrounds and confirming by selection on plates and PCR analysis.

*ura3* reversion strains were constructed in the yeast strain ySR128 (Roberts et al., 2012), which contains *ADE2*, *URA3*, and *CAN1* deleted from their normal chromosomal positions and re-inserted as an array into the *LYS2* gene on chromosome 2. The AA to GT mutations were generated within *URA3* by transformation of duplex oligonucleotides (forward, 5′- AGGCATTATCCGCCAAGTACAATTTTTTACTCTTCGAAGACAGAGTATTTGCTGACATTGGTAATACAGTCAAATTGCAGTACTCTGCGG −3′ and reverse, 5′- CCGCAGAGTACTGCAATTTGACTGTATTACCAATGTCAGCAAATACTCTGTCTTCGAAGAGTAAAAAATTGTACTTGGCGGATAATGCCT-3′) into yeast and selection for ura3 deficient yeast on SC media containing 5-fluoroorotic acid (5-FOA). Proper editing of *URA3* within these isolates was confirmed by isolating total genomic DNA from the strains, PCR amplifying the *ura3* gene, and Sanger sequencing the resulting PCR product. Strains containing only the intended AA to GT tandem substitution and a second benign substitution were subsequently diploidized by transformation of the yeast with the YEpHO plasmid (Murray and Szostak, 1983) that results in expression of the HO endonuclease. HO expression subsequently induces mating type switching in these yeast and allows mating of MATa and MATα yeast. Diploid yeast were isolated by ability of yeast to proliferate on leucine dropout media (a functional *LEU2* gene is encoded on the YEpHO plasmid) and subsequent screening for non-mating Leu+ isolates. A *trp5* E50V point mutant was generated in BY4741 using Cas9 genome editing (Laughery et al., 2015; Laughery and Wyrick, 2019). Briefly, oligonucleotides (forward, 5′- GATCTGGTGTAGATATCATCGAATGTTTTAGAGCTAG-3′ and reverse, 5′-CTAGCTCTAAAACATTCGATGATATCTACACCA-3′) targeting the E50 codon of *TRP5* were hybridized and ligated into pTO40. The resulting plasmid was then transformed into BY4741 along with pJH001 and a template oligonucleotide OHSM001 (CTATTCTCAAGGGTTTCCAGGATGGTGGTGTAGATATCATCGTGTTAGGTATGCCCTTCTCTGATCCAATTGCAGATGGTCCTACAATTC), which, upon recombination resulted in a GAA>GTG mutation in addition to inactivation of the downstream PAM sequence. The *trp5* E50V mutant was confirmed by DNA sequencing.

#### Yeast UV Exposures

Diploid yeast cultures were grown to late log phase in YPD medium. Cells were collected via centrifugation and resuspended in sterile water to an approximate cell density of 1 × 10^7^ cells/mL. 3–5 μL aliquots of resuspended yeast were independently spotted into arrays on YPD plates. Plates were exposed to 12.5 J/m^2^ (*rad16*Δ) or 25 J/m^2^ (wild-type and *rad26*Δ) of UVC radiation and then incubated in the dark for about 2 to 3 days at 30þC. Cells from each individual spot were then re-suspended in sterile water to ~1 × 10^7^ cells/mL or less, re-spotted to fresh YPD plates, and irradiated again at the same dose. After a total of either 9 or 15 UV exposures, a single colony from each independent spot on the arrayed YPD plate was isolated.

#### UV Survival Assay

To assess the survival of *rad16*Δ, *rad26*Δ, and wild-type (BY4741) haploid strains, cultures were grown to mid to late log phase in YPD medium and OD_600_ readings were obtained to determine approximate cell density. Cells were then harvested by centrifugation and resuspended in sterile Millipore water. Serial dilutions were made in sterile water and plated on YPD medium. They were then exposed to approximately 0, 12.5, 25, or 50 J/m^2^ of UVC radiation and put into a dark 30þC incubator. Colony counts were made approximately every 2–4 days and final counts were determined after 8–10 days of incubation.

#### Whole Genome Sequencing of UV-irradiated Yeast

Each independent colony obtained from serial UV exposure was inoculated into fresh liquid YPD and incubated at 30þC overnight. Yeast from these cultures were collected by centrifugation and the genomic DNA was isolated via Phenol:Chloroform:Isoamyl alcohol extraction and ethanol precipitation, adapted from our previously described protocol (Mao et al., 2016). Total genomic DNA was fragmented using a Covaris E220 and libraries for each independent isolate generated using a KAPA DNA HyperPrep kit. Libraries were created for >150 independent isolates. Multiplexed whole genome sequencing of these libraries was conducted on two lanes of an Illumina HiSeq4000. Paired end 150 nt sequencing reads for each isolate where mapped to the Saccer3 S288C reference genome using CLC genomics workbench version 7.5. This resulted in 50–100 fold average coverages for most yeast isolates. Mutations were identified from these alignments similarly to previously published methods (Mao et al., 2017). Mutations acquired during repeated UV exposure were identified as variants in comparison to the reference genome that are supported by greater than 45% of reads covering the site and that occur only within a single isolate of those sequenced.

#### Analysis of Genomic Mutation Data in Yeast

The genomic mutation calls from UV exposed yeast were analyzed by custom perl scripts to separate single, double, and the occasional triple nucleotide substitutions from insertion/deletion (indel) mutations in each isolate. To confirm that the double substitutions were indeed tandem substitutions on the same chromosome, the original mapped sequenced reads were manually inspected for a subset of double mutations. In all cases, both mutations in a tandem substitution occurred in the same chromosome allele. Single nucleotide substitutions were classified according to the pyrimidine-containing DNA strand (i.e., C or T). Custom perl scripts were used to classify mutations by trinucleotide context and mutation class. Mutations occurring in mitochondrial DNA were excluded. Significant differences in the number of mutations in each mutation class per isolate in WT relative to repair-deficient strains were determined using a t test with the Holm-Sidak correction for multiple hypothesis testing. Transcriptional asymmetry of UV mutations was calculated using custom perl scripts for the transcribed regions of ~5000 genes, using published transcription start sites (TSS) and transcription end sites (TES) in yeast (Park et al., 2014), and normalized based on the frequency of each trinucleotide in the NTS and TS of each yeast gene, excluding overlapping gene regions on opposite strands. Genes overlapping with repetitive rDNA (i.e., YLR154C-G, YLR155C, YLR161W, YLR162W-A) and *CUP1* (i.e., YHR053C, YHR054C, YHR055C) loci were also excluded. Significant transcriptional asymmetry, as reflected by differences in the number of mutations on the TS relative to the NTS, was determined using a chi-square test and Bonferroni correction for multiple hypothesis testing. Only trinucleotide mutation classes with at least 30 mutants were included in this analysis. For analysis of double mutations, only double mutant classes with at least 19 mutants were tested for transcriptional asymmetry.

#### CAN1 Reporter Assay

To estimate the abundance of UV-induced mutations occurring on a genomic scale, *CAN1* mutation assays were performed on *rad16*Δ, *rad26*Δ, and wild-type (WT) haploid strains. Cultures were grown until mid to late log phase in YPD medium, collected by centrifugation, and resuspended in sterile Millipore water. They were then poured into Petri dishes and exposed to approximately 12.5 J/m^2^ (*rad16*Δ and WT) or 25 J/m^2^ (WT and *rad26*Δ) of UVC radiation in a dark room. Control cells were also poured into Petri dishes, but not exposed to UVC. All cells were then centrifuged, decanted, and resuspended in fresh YPD medium. Afterward, they were incubated with shaking overnight at 30þC in the dark. The next day, OD_600_ readings were used to determine cell concentration, and appropriate dilutions were made in PBS and plated on SC-Arg+Can and SC medium. Plates were incubated about 3–4 days before counting colonies. UV-induced *CAN1* mutation frequencies were determined from the number of canavanine resistant (Can^R^) colonies using the equation below, as previously described (Hoopes et al., 2016, 2017; Laughery et al., 2019; Mao et al., 2017).

$$\text{Mutation Freq}.=\frac{\left(\#\text{of }{\text{Can}}^{R}\text{ colonies}\right)\left(\text{Dilution for Canavanine plates}\right)}{\left(\#\;\text{ofcolonies on SC plate}\right)\left(\text{Dilution for SC plates}\right)}$$

#### URA3 Reversion Assay

To assess whether UV light could revert the *ura3* K93V mutant, yeast cultures were grown in YPD until mid to late log phase and harvested via centrifugation. The pellets were resuspended in sterile Millipore water to make a stock cell suspension and aliquots were spread on plates lacking uracil (SC-Ura) at an approximate density of 1 × 10^7^ – 1×10^8^ cells/plate. Serial dilutions were also made of the stock cell suspension and plated on SC plates to calculate the actual number of cells plated. Plates were then exposed to approximately 50 J/m^2^ of UVC or 300 J/m^2^ UVB irradiation or left unexposed (control group) and incubated in the dark at 30þC for approximately 4–5 days. Colonies as well as ambiguous growth were patched to SC- Ura plates and incubated at 30þC to confirm viability on SC-Ura medium. Control colonies in which more than half the area of growth was on the edge of the plate were discarded from total cell number calculations, however all revertant colonies found on a plate were counted. URA+ reversion mutations were confirmed by sequencing the *URA3* gene and PCR amplifying a DNA fragment between the *ADE2* and *URA3* genes to ensure URA+ isolates were derived from the originating yeast strain and contained the expected GT to AA substitution.

#### TRP5 Reversion Assay

To test the reversion frequency of the *trp5* E50V mutant upon irradiation with UV light, cultures were grown in YPD medium until mid or late log phase and harvested via centrifugation. After decanting the media, cell pellets were resuspended in sterile Millipore water to make a stock cell suspension and then spread onto plates lacking tryptophan (SC-Trp) at an approximate density of 5×10^7^ cells/plate. Serial dilutions were also made from the stock suspension and plated on synthetic complete (SC) plates to calculate the actual amount of cells plated. Plates were then exposed to ~25 J/m^2^ UVC light, 300 J/m^2^ UVB light, or left unexposed (control group) in a dark room, after which they were incubated at 30°C in the dark. Colonies were counted after about 3 days, and ambiguous growth was re-patched to SC-Trp plates and allowed to grow at 30þC to reassess viability on medium lacking tryptophan. Control colonies in which more than half the area of growth was on the edge of the plate were discarded from total cell number calculations; however, all revertant colonies found on a plate were counted. For yeast treated with 0 J/m^2^ UVC, only 3 TRP+ revertants were recovered in total. Therefore, a maximum estimated frequency was calculated as if each replicate contained at least one TRP+ colony. A replicate trial of the “No UV experiment” for the UVB reversion assay had a similarly low number of only 8 TRP+ revertants out of ~17 billion cells plated. TRP+ reversion mutants were analyzed by isolating genomic DNA from TRP+ colonies, PCR amplifying the mutation locus with Phusion polymerase (NEB), and sequencing. The presence of the neighboring SNP resulting from strain construction was used to confirm the strain background.

#### *In vitro* characterization of TA photoproduct

The following oligos were used for UVDE digestion of UV-induced TA photoproducts:

Forward Primer 5′-GCGTGTGCACGTATATATATACGCGCGTGTG-3′ and

Reverse Primer 5′Biotin-CACACGCGCGTATATATATACGTGCACACGC-5′.

Annealed oligos were labeled with [γ^32^P]ATP (Perkin Elmer) using T4 polynucleotide kinase (NEB, M0201L). The labeled primers were purified using Illustra Microspin G-50 column (GE healthcare) and 40 μL of the purified, labeled DNA was spotted onto glass coverslips as four spots (10 μl each) for each dose and exposed to UVC light using UV StratalinkerTM 1800. The spots were recovered from the coverslip and ethanol precipitated. The damaged DNA samples were digested with UVDE enzyme in a reaction buffer (pH 6.5) containing HEPES (20mM), NaCl (100mM), and MnCl_2_ (1mM) at 55þC for 1 hour. Digested DNA was then ethanol precipitated, washed and dissolved in 5 μL of deionized water. Formamide was added to the samples to a final concentration of 50% and heated at 80þC for 5 minutes and loaded on to a pre-run 15% denaturing polyacrylamide urea gel. The gel was run for 2 hours and 10 minutes. The gel was exposed to phosphor screen and the radioactivity signal was imaged using a Typhoon scanner (GE Healthcare). The TA lesion band intensity was quantified using ImageQuant TL software.

#### UVDE cloning, expression, and isolation

The amino acid sequence of *T. thermophiles* UVDE was obtained from UniProtKB (ID# Q746K1) and reverse translated into a cDNA sequence with optimal codon usage for expression in *E. coli*. The resulting DNA sequence was synthesized as a geneblock (Integrated DNA Technologies) containing NcoI and BamHI restriction sites appended onto the 5′ and 3′ portions of the coding sequence, respectively. Both the geneblock and the pETHT bacterial expression vector were digested with NcoI and BamHI and gel purified using a QIAGEN gel extraction kit. The digested gene block was directionally ligated into the multiple cloning sequence of the pETHT bacterial expression vector (Brady et al., 1996) using T4 DNA ligase (NEB, M0202T) to generate an N-terminal 6xHis-tagged UVDE protein. Ligation reactions were transformed into DH5α *E. coli* and successfully generated UVDE expression plasmids selected by growth of transformed *E. coli* on LB plates containing ampicillin. The UVDE cDNA within the selected plasmids were verified by Sanger sequencing prior to transformation of the expression vector into BL21 pLysS *E. coli* cells (Thermo Fisher Scientific, C606003). BL21 cells containing the UVDE expression vector where expanded at 30°C to a 1 L culture in LB medium until obtaining an O.D. of 1 after which ITPG was added to a final concentration of 0.5 mM to induce UVDE expression for 4 hr. *E. coli* were harvested by centrifugation, re-suspended in 20 mL of extraction buffer (20 mM Tris-HCl pH 7.5, 500 mM NaCl, 20 mM imidizole, 10 mM β-mercaptoethanol, 0.5% Trition X-100, 0.5% Tween 20, 10% glycerol, 2X EDTA-free protease inhibitors, and 35 mg lysozyme), and sonicated 6 times for 30 s each using a Misonix Sonicator 3000 with a microtip set to a power of 5. Crude lysates where clarified by centrifugation for 20 min at 15K rpm in a Sorvall centrifuge using a SS-34 rotor. Remaining particulates were removed by filtration. 6xHis-tagged UVDE was purified by flowing the extract over a column of Ni^2+^ Sepherose 6 Fast Flow media (GE Biosciences) equilibrated in extraction buffer. Following binding of the UVDE to the column, the column was washed successively with 5 column volumes of extraction buffer and 5 column volumes of an additional buffer containing lower levels of salt and detergent (20 mM Tris-HCl pH 7.5, 25 mM KCl, 20 mM imidizole,10 mM β-mercaptoethanol, 0.1% Trition X-100, and 10% glycerol). UVDE was eluted in 20 mM Tris-HCl pH 7.5, 25 mM KCl, 250 mM imidizole, 10 mM β-mercaptoethanol, 0.1% Trition X-100, and 10% glycerol. The UVDE was further purified by pooling the eluted fractions and applying them to a Q Sepharose Fast Flow (GE Biosciences) column equilibrated in 20 mM Tris-HCl pH 7.5, 25 mM KCl, 1 mM DTT, 0.1% Trition X-100, and 10% glycerol. UVDE was subsequently eluted from the Q column over a gradient to 1M NaCl over 25 column volumes. Fractions containing UVDE were pooled and flash frozen in liquid nitrogen for future use in the UVDE-seq protocol.

#### CPD photolyase cloning, expression, and isolation

The amino acid sequence of the *E. coli* CPD photolyase was obtained from UniProtKB (accession# P00914; gene name *phrB*), reverse translated and codon optimized. A geneblock of the resulting sequence was synthesized containing 5′ NdeI and 3′ BamHI restriction sites (Integrated DNA Technologies) and directionally cloned into the corresponding NdeI and BamHI sites of the pET16b bacterial expression vector as previously described for the cloning of UVDE. The resulting vector encoding a 10XHis tagged CPD photolyase was sequence confirmed and transformed into T7 Express *lysY E. coli* (NEB, C3010I). To avoid bacteria entering stationary phase and reducing *phrB* expression, a single colony containing the *phrB* expression vector was expanded to a 1 L culture, by inoculating the colony into 5 mL of LB media containing ampicillin and growing at 37°C for 8 hr. 1 mL of this culture was then diluted into 100 mL of LB containing ampicillin and grown overnight at 37°C. The following morning, 10 mL of the overnight culture was diluted into 1 L of LB containing ampicillin and grown at 37°C to and OD between 0.6 and 0.8. IPTG was added to a concentration of 0.5 mM and the culture grown an additional 4 hr at 37°C to induce *phrB* expression. Cells expressing *phrB* were then harvested by centrifugation, re-suspended in 25 mL of lysis buffer (50mM HEPES pH 7, 50mM Dextrose, 0.5M NaCl, 20 mM Imidizole, 10 mM β-mercaptoethanol, 2X EDTA-free protease inhibitors, and 40 mg lysozyme). Cells were lysed and a clarified supernatant obtained as described for the purification of UVDE. The CPD photolyase containing clarified lysate was bound to a 1 mL HisTrap column (GE Biosciences) previously equilibrated in lysis buffer. The HisTrap column was washed with 5 column volumes of lysis buffer, followed by an additional 5 column volumes of a lower salt wash buffer (50mM HEPES pH 7, 100mM KCl, 20 mM Imidizole, 10 mM β-mercaptoethanol). CPD photolyase was eluted from the Ni^2+^ column with 50mM HEPES pH 7, 100mM KCl, 500 mM Imidizole, 10 mM β-mercaptoethanol. The resulting eluate was then bound to a 1 mL HiTrap Blue column (GE Biosciences) equilibrated in 50mM HEPES pH 7, 100mM KCl, 10 mM DTT and subsequently eluted using a 50 mL gradient to 50mM HEPES pH 7, 2M KCl, 10 mM DTT. Fractions containing CPD photolyase were pooled and dialyzed to 50 mM Tris 8, 50mM NaCl, 0.1 mM EDTA, 10 mM DTT, 50% glycerol for storage.

#### UVDE-seq library preparation and sequencing

We adapted the emRiboSeq protocol (Ding et al., 2015) and our yeast CPD-seq protocol (Mao et al., 2016) to create the UVDE-seq method. Haploid WT and *rad16*Δ cells were grown in YPD to an OD600 ~0.8. Cells were then pelleted and re-suspended in sterile Millipore water. Cells were taken for the “No UV” control group, while the remaining cells were exposed to 600 J/m^2^ UVC light. Cells were then pelleted and stored at −80C until genomic DNA was isolated using phenol:chloroform:isoamylalcohol (PCI), lysis buffer, and glass beads. Purified *S. cerevisiae* genomic DNA was sonicated in a Bioruptor 300 Sonicator (Diagenode, UCD-300 TM) for 15 cycles (30 s ON/OFF intervals) to create fragments between 200 and 500bp in length. DNA fragments were then end-repaired (NEB, E6050L) and dA-tailed (NEB, E6053L), and a double stranded trP1 adaptor was ligated to both ends of the fragments via a quick ligase module (NEB, E6056L). trP1 adaptor ligation was confirmed by PCR using primers complimentary to the trP1 adaptor. Following confirmation, free 3′-OH groups were blocked with Terminal Transferase (NEB, M0315L) either dideoxyATP or dideoxyGTP (Roche Diagnostics, 03732738001). Samples were then treated with *E. coli* CPD photolyase, and incubated under 365nm UV light for 2 hours at room temperature. DNA fragments were purified using a phenol:chloroform:isoamylalcohol (PCI) extraction, followed by ethanol precipitation. Samples were then treated with *T. thermophilus* UVDE for 45 minutes at 55°C. 5′ phosphate groups were removed using shrimp alkaline phosphatase (Affymetrix, AF78390500), and DNA was subsequently denatured at 95°C for 5 minutes and snap-cooled on ice. A second double stranded adaptor, the A adaptor, was then ligated to the 3′-OH created immediately downstream of the cleaved UV lesion (NEB, E6056L). Second adaptor ligation was PCR confirmed using a Cy3-labeled primer complimentary to the A adaptor. Each A adaptor contains a unique barcode that allows for the creation of different libraries to be pooled and analyzed through multiplexed DNA sequencing techniques, as well as a biotin-labeled strand. DNA containing the biotin label was purified using Streptavidin beads (Thermo Fisher Scientific, 11205D), while the DNA strand lacking the biotin label was removed using 0.15M NaOH. The remaining ssDNA was then used as a template for second strand synthesis, using the second strand of adaptor A as the extension primer. Libraries were PCR amplified for 8 cycles using trP1 and A primers. Finally, samples were combined at equal volumes and submitted for Ion Proton sequencing (Life Technologies). The resulting UVDE-seq reads were mapped to the yeast genome using bowtie2 (Langmead and Salzberg, 2012), and the corresponding dinucleotide damage site was identified and counted, essentially as previously described (Mao et al., 2016).

#### Analysis of Somatic Mutations in Cutaneous Melanomas and cSCCs

Melanoma mutation data were obtained from the ICGC data portal (data release 20), yielding a total of 140 cutaneous melanoma tumor genomes. Cutaneous squamous cell carcinoma (cSCC) mutation data from *XPC*^−/−^ individuals were from (Zheng et al., 2014). Mutation data were processed as previously described (Mao et al., 2018; Mao et al., 2020). Classification of mutations by trinucleotide context and mutation class were performed as described for yeast mutations. Transcriptional asymmetry of UV mutations was calculated using custom perl scripts for the transcribed regions of human genes, defined by GENCODE (version 10, hg19) (Harrow et al., 2012). Transcriptional asymmetry was computed separately for the top and bottom expressed quartile of genes based on Roadmap Epigenome RPKM normalized RNA-seq data (Kundaje et al., 2015) for melanocytes (E059) and keratinocytes (E057) (obtained from https://egg2.wustl.edu/roadmap/data/byDataType/rna/expression/) for cutaneous melanoma and cSCC, respectively, and normalized based on the frequency of each trinucleotide in the NTS and TS of each gene set. Significant transcriptional asymmetry, as reflected by differences in the number of mutations on the TS relative to the NTS, was determined using a chi-square test and Bonferroni correction for multiple hypothesis testing. For analysis of double mutations, only double mutant classes with at least 1000 mutants were tested for transcriptional asymmetry. Mutation data from 35 sequenced acral melanoma genomes (ICGC data portal data release 20) were used for comparison with cutaneous melanomas. Analysis of mutations in the coding exons of driver genes used driver genes identified in two recent papers (Hayward et al., 2017; Hodis et al., 2012), namely ARID2, BRAF, CDK4, CDKN2A, DDX3X, GNAQ, HRAS, KIT, KRAS, MAP2K1, MAP2K2, NF1, NRAS, PPP6C, PTEN, RAC1, RASA2, RB1, SF3B1, SNX31, STK19, TACC1, and TP53. Coding exons identified from the UCSC Table Browser were used for identifying driver mutations in the cutaneous melanoma dataset.

#### Analysis of BRAF and NRAS mutations in human cancers

The mutational status of the *BRAF* gene in 265047 human cancers and *NRAS* gene in 100375 human cancers were obtained from the COSMIC database (https://cancer.sanger.ac.uk/cosmic/gene/analysis?ln=BRAF; https://cancer.sanger.ac.uk/cosmic/gene/analysis?ln=NRAS). Samples that had an unspecified tissue of origin, an ambiguous nucleotide change annotation, or that were duplicated were removed from subsequent analysis. The number of instances of each substitution mutation observed in the dataset was determined. For each mutation occurring at least 10 times in the dataset an odds-ratio for its representation among human skin cancers was calculated as the ratio of skin cancer samples containing the mutation to skin cancers without the mutation divided by the ratio of non-skin cancers that contain the mutation to non-skin cancers without the mutation. Similarly, the incidence of specific *BRAF* mutations in skin and thyroid cancers were compared by calculating odds-ratios for mutations occurring at least 10 times in either of these two cancer types. Odds-ratios were calculated as the ratio of the number of skin cancer samples with a mutation to the number of skin cancers without the mutation divided by the ratio of number of thyroid cancers with a mutation to thyroid cancers without the mutation.

### QUANTIFICATION AND STATISTICAL ANALYSIS

All quantification of mutation and UV-induced photoproduct abundances utilized custom perl scripts. Subsequent statistical evaluation of these abundances and comparison of mutation frequencies were calculated using GraphPad Prism software. Statistical methods used and replicates evaluated are indicated in the legends of relevant figures as well as in the Method Details. A minimal statistical significance threshold of p < 0.05 was utilized for analyses. For analyses with multiple hypothesis testing correction, a q < 0.05 threshold of significance was employed.

## Supplementary Material

1

2

3

## Figures and Tables

**Table:** KEY RESOURCES TABLE

REAGENT or RESOURCE	SOURCE	IDENTIFIER
Bacterial and Virus Strains		
*E. coli*: BL21 pLysS	Thermo Fisher Scientific	Cat#C606003
*E. coli*: T7 Express	New England BioLabs	Cat#C3010I
*E. coli*: DH5α	Thermo Fisher Scientific	Cat# 18258012
Chemicals, Peptides, and Recombinant Proteins		
T4 polynucleotide kinase	New England BioLabs	Cat# M0201L
T4 DNA ligase	New England BioLabs	Cat#M0202T
NEBNext End Repair Module	New England BioLabs	Cat#E6050L
NEBNext dA-Tailing Module	New England BioLabs	Cat#E6053L
Terminal Transferase	New England BioLabs	Cat#M0315L
UVDE	This Study	NA
CPD photolyase	This Study	NA
shrimp alkaline phosphatase	Affymetrix	Cat#AF78390500
NEBNext® Quick Ligation Module	New England BioLabs	Cat#E6056L
Streptavidin beads	Thermo Fisher Scientific	Cat#11205D
Critical Commercial Assays		
KAPA DNA HyperPrep	Roche Scientific	Cat# 07962363001
Deposited Data		
Saccer3 yeast reference genome	Saccharomyces Genome Database	https://hgdownload.soe.ucsc.edu/goldenPath/sacCer3/bigZips/sacCer3.fa.gz
Yeast transcription start and end sites	(Park et al., 2014)	NA
Melanoma Mutation Data	International Cancer Genome Consortium	https://dcc.icgc.org/api/v1/download?fn=/release_20/Projects/MELA-AU/simple_somatic_mutation.open.MELA-AU.tsv.gz
Cutaneous squamous cell carcinoma mutation data	(Zheng et al., 2014)	NA
RPKM normalized RNA-seq data for melanocytes (E059)	Roadmap Epigenome	https://egg2.wustl.edu/roadmap/data/byDataType/rna/expression/
RPKM normalized RNA-seq data for keratinocytes (E057)	Roadmap Epigenome	https://egg2.wustl.edu/roadmap/data/byDataType/rna/expression/
GENCODE (version 10, hg19)	(Harrow et al., 2012)	NA
Mutational status of the *BRAF* gene	COSMIC Database	https://cancer.sanger.ac.uk/cosmic/gene/analysis?ln=BRAF
Mutational status of the NRAS gene	COSMIC Database	https://cancer.sanger.ac.uk/cosmic/gene/analysis?ln=NRAS
UVDE-seq	This Study	GEO: GSE144679
Yeast whole genome sequencing	This Study	SRA: PRJNA605561
List of UV-induced mutations in yeast	This Study	Data S1
Experimental Models: Organisms/Strains		
S. cerevisiae: BY4741 (Wild Type) *MATa his3Δ1 leu2Δ0 met15Δ0 ura3Δ0*	Dharmacon, Inc	Cat#YSC1048
S. cerevisiae: BY4742 (Wild Type): *MATα his3Δ1 leu2Δ0 lys2Δ0 ura3Δ0*	ATCC	Cat#201389
S. cerevisiae: MP072 (*rad16*Δ): *MATa his3Δ1 leu2Δ0 met15Δ0 ura3Δ0 trp1::HIS3 rad16::TRP1*	(Mao et al., 2017)	NA
S. cerevisiae: MP071 (*rad26*Δ): *MATa his3Δ1 leu2Δ0 met15Δ0 ura3Δ0 trp1::HIS3 rad26::TRP1*	(Mao et al., 2017)	NA
S. cerevisiae: YML153 (*rad16*Δ): *MATα his3Δ1 leu2Δ0 lys2Δ0 ura3Δ0 rad16::LEU2*	This Study	NA
S. cerevisiae: YML150 (*rad26*Δ): *MATα his3Δ1 leu2Δ0 lys2Δ0 ura3Δ0 rad26::LEU2*	This Study	NA
S. cerevisiae: BY4743 (Wild Type diploid): *MATa/α his3Δ1/his3Δ1 leu2Δ0/leu2Δ0 LYS2/lys2Δ0 met15Δ0/MET15 ura3Δ0/ura3Δ0*	Dharmacon, Inc	Cat#YSC1050
S. cerevisiae: YML155 (*rad16*Δ diploid): *MATa/α his3Δ1/his3Δ1 leu2Δ0/leu2Δ0 LYS2/lys2Δ0 met15Δ0/MET15 ura3Δ0/ura3Δ0 TRP1/trp1::HIS3 rad16::TRP1/rad16::LEU2*	This Study	NA
S. cerevisiae: YML152 (*rad26*Δ diploid): *MATa/α his3Δ1/his3Δ1 leu2Δ0/leu2Δ0 LYS2/lys2Δ0 met15Δ0/MET15 ura3Δ0/ura3Δ0 TRP1/trp1::HIS3 rad26::TRP1/rad26::LEU2*	This Study	NA
S. cerevisiae: ySR185 (WT diploid): *MATa/α his7-2/his7-2 ura3Δ/ura3Δ can1Δ/can1Δ ade2Δ/ade2Δ leu2-3,112/leu2-3,112 trp1-289/trp1-289 lys2::ADE2-URA3-CAN1/lys2::ADE2-URA3-CAN1*	This Study	NA
S. cerevisiae: YHSM1 (*trp5* E50V): *MATa his3Δ1 leu2Δ0 met15Δ0 ura3Δ0 trp5-E50V*	This Study	NA
S. cerevisiae: ySR128 (Wild Type): *MATα his7-2 ura3Δ can1Δ ade2Δ leu2-3,112 trp1-289 lys2::ADE2-URA3-CAN1*	(Roberts et al., 2012)	NA
S. cerevisiae: DM01 (*ura3* K93V): *MATα his7-2 ura3Δ can1Δ ade2Δ leu2-3,112 trp1-289 lys2::ADE2-ura3-K93V-CAN1*	This Study	NA
S. cerevisiae: DM02 (*ura3* K93V): *MATα his7-2 ura3Δ can1Δ ade2Δ leu2-3,112 trp1-289 lys2::ADE2-ura3-K93V-CAN1*	This Study	NA
S. cerevisiae: DM03 (*ura3* K93V diploid): *MATa/α his7-2/his7-2 ura3Δ/ura3Δ can1Δ/can1Δ ade2Δ/ade2Δ leu2-3,112/leu2-3,112 trp1-289/trp1-289 lys2::ADE2-URA3-CAN1/lys2::ADE2-ura3-K93V-CAN1*	This Study	NA
S. cerevisiae: DM04 (*ura3* K93V diploid): *MATa/α his7-2/his7-2 ura3Δ/ura3Δ can1Δ/can1Δ ade2Δ/ade2Δ leu2-3,112/leu2-3,112 trp1-289/trp1-289 lys2::ADE2-URA3-CAN1/lys2::ADE2- ura3-K93V-CAN1*	This Study	NA
Oligonucleotides		
Primers: see Table S3	This Study	NA
Recombinant DNA		
*T. thermophiles* UVDE cDNA	UniProtKB	ID#Q746K1
*E. coli* CPD photolyase; gene name *phrB*	UniProtKB	ID#P00914
pETHT	(Brady et al., 1996)	NA
pET16b	MilleporeSigma	Cat#69662
pAB001	This Study	NA
pET16b-CPDphotolyase	This Study	NA
YEpHO	(Murray and Szostak, 1983)	NA
pRS405	ATCC	Cat#87516
Software and Algorithms		
CLC genomics workbench version 7.5	QIAGEN	Cat#832001
ImageQuant TL	GE Biosciences	Cat#29000737
bowtie2	(Langmead and Salzberg, 2012)	http://bowtie-bio.sourceforge.net/bowtie2/index.shtml
GraphPad Prism 6	GraphPad Software	https://www.graphpad.com/

## Highlights

- Genome sequencing of UV-irradiated yeast reveals non-canonical mutation classes
- Non-canonical mutations are likely caused by atypical UV photoproducts
- UV induces an atypical thymine-adenine (TA) photoproduct *in vitro* and in yeast cells
- Non-canonical UV mutation classes can explain many driver mutations in melanoma
