# Supplementary material for: Atypical UV Photoproducts Induce Non-canonical Mutation Classes Associated with Driver Mutations in Melanoma
Source: Cell Rep. Author manuscript; Available in PMC 2020 Dec 2. (PMC7709870; doi:10.1016/j.celrep.2020.108401)
Supplement: 1 [file NIHMS1647633-supplement-1.pdf]

Cell Reports, Volume *33*

Supplemental Information

Atypical UV Photoproducts

Induce Non-canonical Mutation Classes

Associated with Driver Mutations in Melanoma

Marian F. Laughery, Alexander J. Brown, Kaitlynne A. Bohm, Smitha Sivapragasam, Haley S. Morris, Mila Tchmola, Angelica D. Washington, Debra Mitchell, Stephen Mather, Ewa P. Malc, Piotr A. Mieczkowski, Steven A. Roberts, and John J. Wyrick

SUPPLEMENTAL INFORMATION

Atypical UV photoproducts induce non-canonical mutation classes associated with driver mutations in melanoma

Marian F. Laughery, Alexander J. Brown, Kaitlynne A. Bohm, Smitha Sivapragasam, Haley S. Morris, Mila Tchmola, Angelica D. Washington, Debra Mitchell, Stephen Mather, Ewa P. Malc, Piotr A. Mieczkowski, Steven A. Roberts, and John J. Wyrick

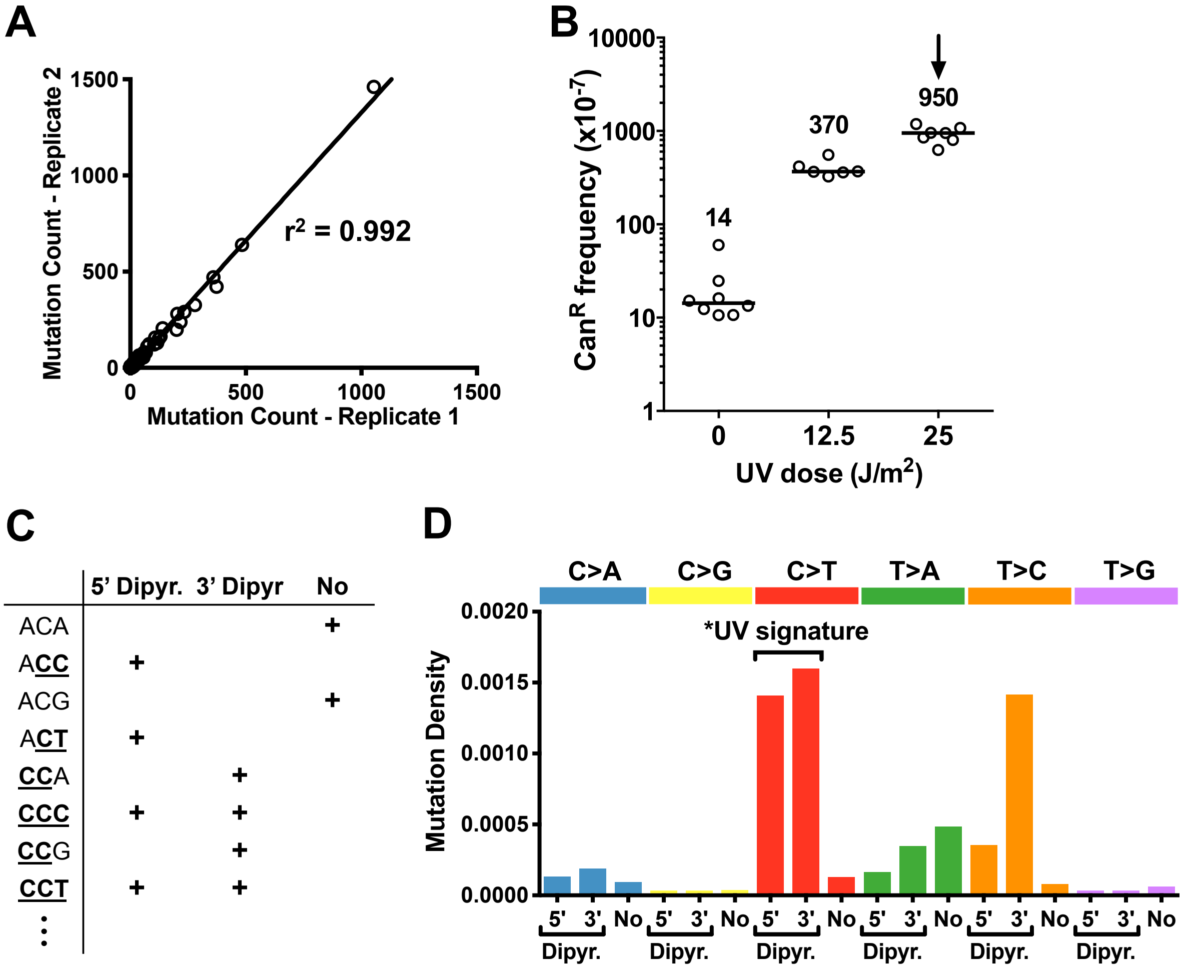

Figure S1: Analysis of UV-induced mutations in yeast. Related to Figure 1. (A) UV mutation spectra in yeast genomes are highly reproducible. Comparison of the numbers of different classes of mutations (e.g., C>A, C>G, etc.) associated with different trinucleotide contexts in replicate 15 dose UV exposure experiments. Each data point indicates a different mutation class and its associated trinucleotide context (e.g., C>T in TCC context; underlined base is mutated). (B) Frequency of canavanine resistant (Can^R) mutant colonies following exposure of wild-type (WT) yeast to a single dose of UVC light. (C) Schematic showing how mutations in different trinucleotide contexts are assigned to categories based on whether the mutation occurs in a dipyrimidine (Dipyr) sequence or not (No). Dipyrimidine-associated mutations are further stratified into 5' Dipyr (i.e., mutation occurs in 5' position of a dipyrimidine) and 3' Dipyr (i.e., mutation occurs in the 3' position of a dipyrimidine). In some trinucleotide contexts (e.g., CCC), a mutation is classified as both a 5' and 3' dipyrimidine. (D) Density of mutations for UV exposed yeast in each mutation class, as defined in panel C. Mutation density was calculated by dividing the mutation count for each class by the number of possible mutable sites for that class in the yeast genome.

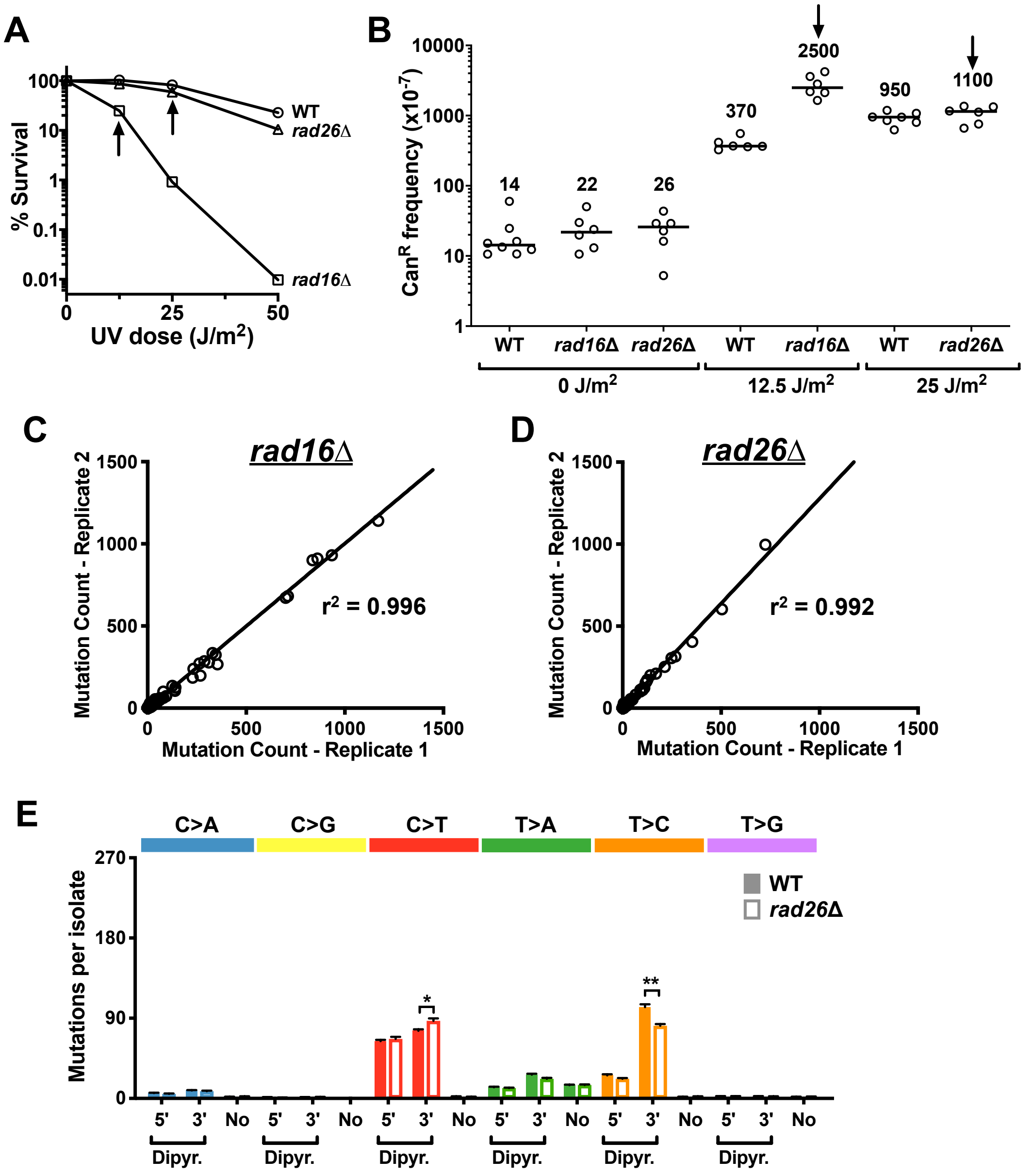

Figure S2: Analysis of UV-induced mutations in repair-deficient *rad16*Δ and *rad26*Δ mutant strains. Related to Figure 2. (A) Survival of WT or NER-deficient yeast cells following UV radiation. (B) UV-induced *CAN1* mutation frequency in WT or NER-deficient strains. The frequency of canavanine-resistant (Can^R) mutants is plotted**.** (C) UV mutation spectra in sequenced *rad16*Δ isolates are highly reproducible. Comparison of the numbers of different classes of mutations (e.g., C>A, C>G, etc.) associated with different trinucleotide contexts in replicate 15 dose UV exposure experiments. Each data point indicates a different mutation class and its associated trinucleotide context (e.g., C>A in TCG context; underlined base is mutated). (D) Same as panel C, except for sequenced *rad26*Δ isolates. (E) Deletion of *RAD26* only slightly alters the spectra of UV-induced mutations. Mean ± SEM is depicted for WT or *rad26*Δ mutant isolates. 'Dipyr.' indicates the mutation occurs at either the 5' or 3' position in a dipyrimidine. Significant differences in the number of mutations in each mutation class per isolate in

WT relative to the *rad26*Δ mutant strain was determined using a *t*-test with the Holm-Sidak correction for multiple hypothesis testing. **$P < 0.001$; *$P < 0.01$.

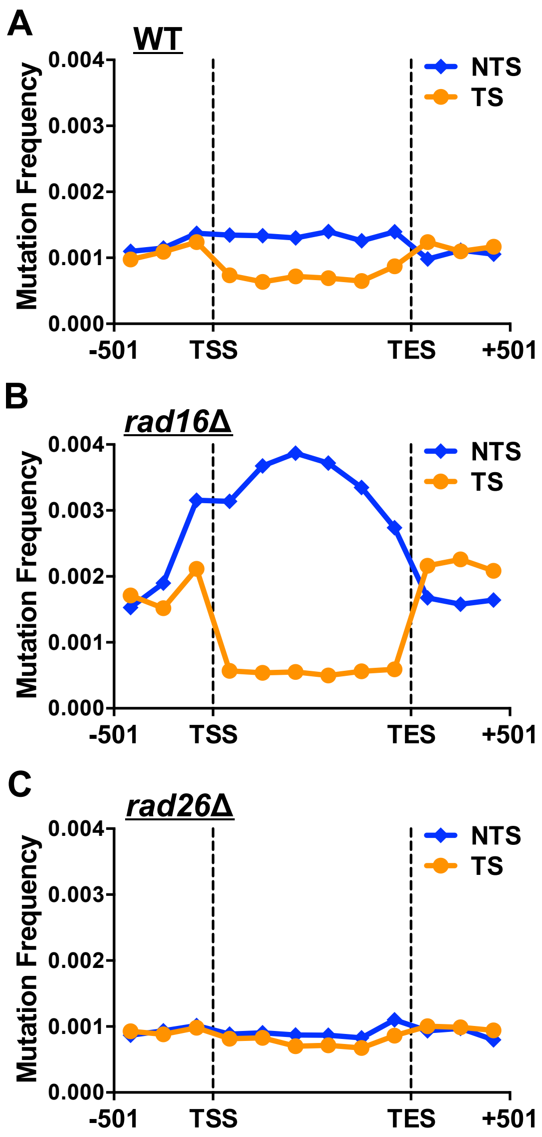

Figure S3: Related to Figure 2. Transcriptional asymmetry in the frequency of single nucleotide substitutions on the transcribed strand (TS) relative to the non-transcribed strand (NTS) for 4971 yeast genes in (A) WT, (B) *rad16*Δ, and (C) *rad26*Δ strains. Each gene is divided into 6 equally sized bins, from the transcription start site (TSS) to the transcription end site (TES), based on published gene coordinates (Park et al., 2014). In addition, 3 bins upstream of the TSS (i.e., -1 to -501 bp) and 3 bins downstream of the TES (+1 to +501 bp downstream of the TES) are also included. Mutations are assigned to the pyrimidine-containing DNA strand.

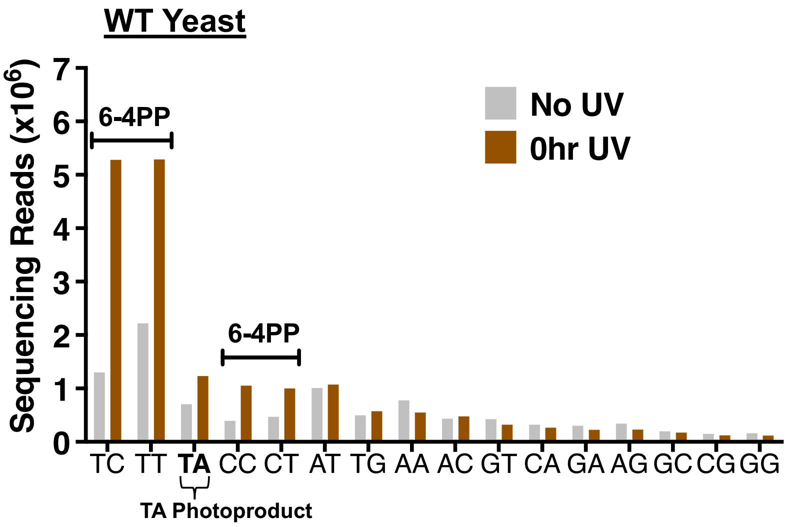

Figure S4: UVDE-seq lesion mapping reveals TA photoproducts in UV-irradiated wild-type (WT) yeast. Related to Figure 3. Number of UVDE-seq reads associated with putative lesion at indicated dinucleotide sequence in UV irradiated WT yeast (600 J/m^2 of UVC light; 0hr UV) and in an unirradiated control strain (No UV).

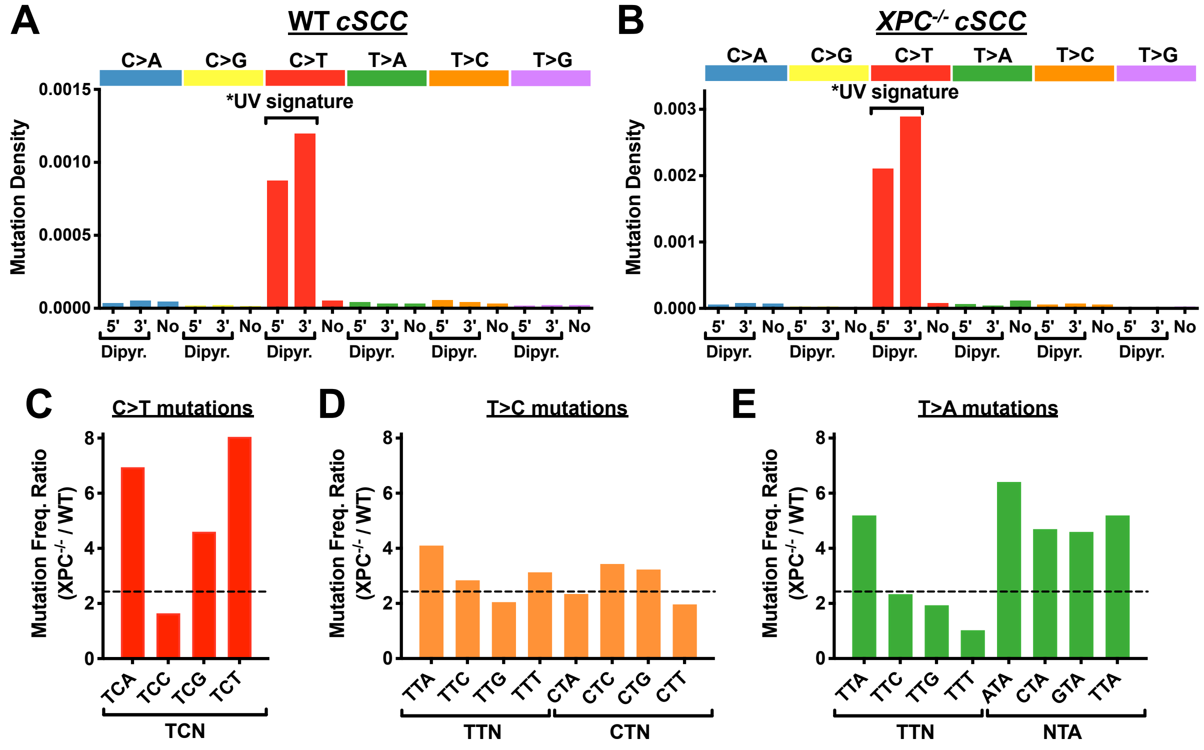

Figure S5: Non-canonical UV mutations (e.g., T>C and T>A) are elevated in repair-deficient *XPC*$^{-/-}$ cutaneous squamous cell carcinomas (cSCCs). Related to Figure 5. (A,B) Density of mutations for WT cSCCs and repair deficient *XPC*$^{-/-}$ cSCCs in each mutation class**.** Mutation density was calculated by dividing the mutation count for each class by the number of possible mutable sites for that class in the human genome. Data from (Zheng et al., 2014). (C-E) Ratio of mutation frequency per tumor for 5 *XPC*$^{-/-}$ cSCCs relative to 8 WT cSCCs. Median ratio across all mutation types is indicated with a dashed line.

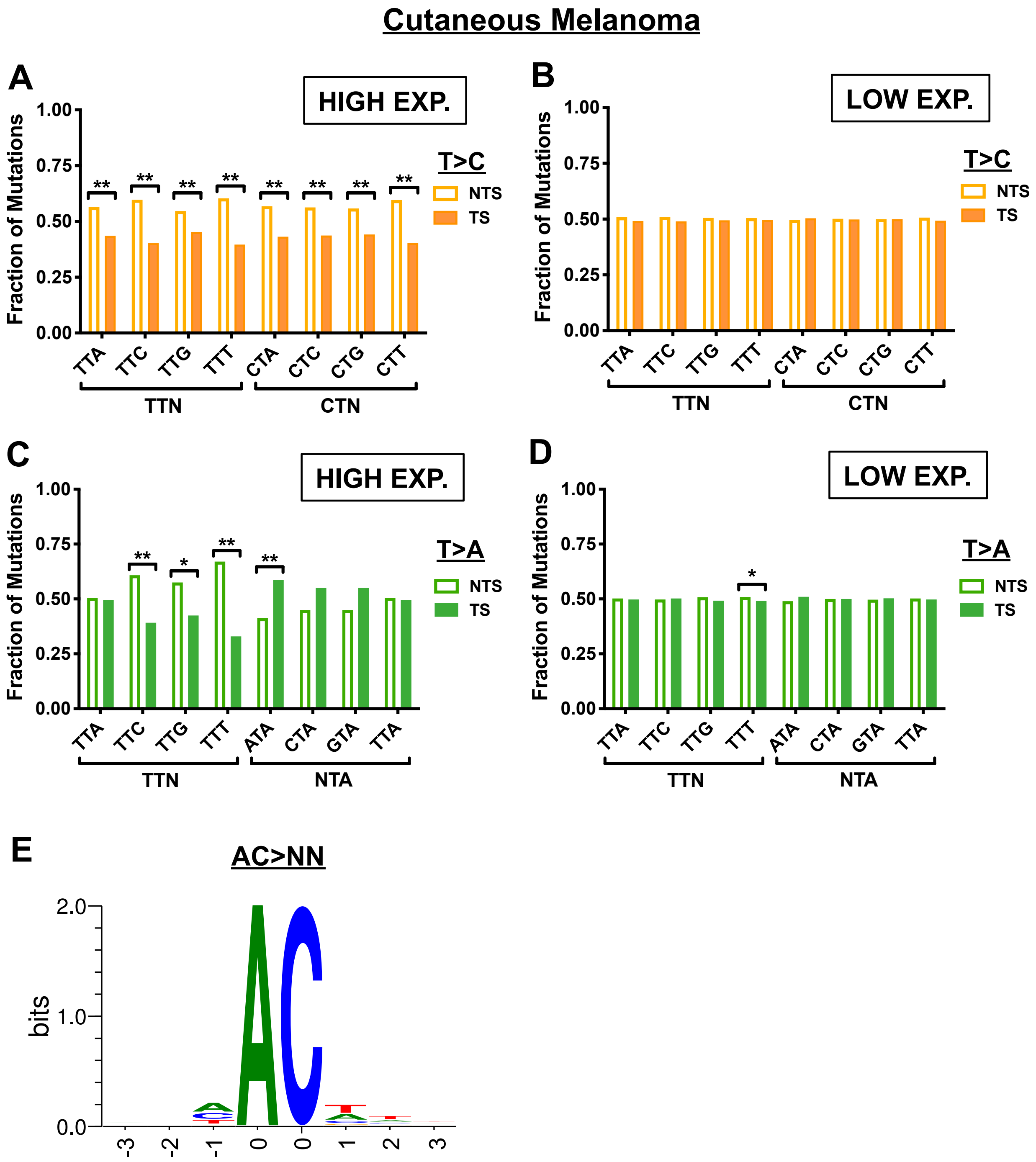

Figure S6: Transcriptional asymmetry of T-to-C (T>C) and T-to-A (T>A) somatic mutation classes in cutaneous melanoma. Related to Figures 5 and 6. (A,B) T>C mutations in dipyrimidine contexts show significant transcriptional asymmetry in genes that are highly expressed in melanocytes (top quartile, panel A), but not in genes that are lowly expressed in melanocytes (bottom quartile, panel B). (C,D) Same as panels A,B, except for T>A mutation classes. Statistical significance was determined using the chi-square test and Bonferroni correction for multiple hypothesis testing. ***P* < 0.001; **P*

< 0.05. (E) Sequence logo representation of DNA flanking all AC>NN tandem substitutions (e.g., AC>TT, AC>CT, etc.) in cutaneous melanomas. Logo was generated using weblogo (Crooks et al., 2004).

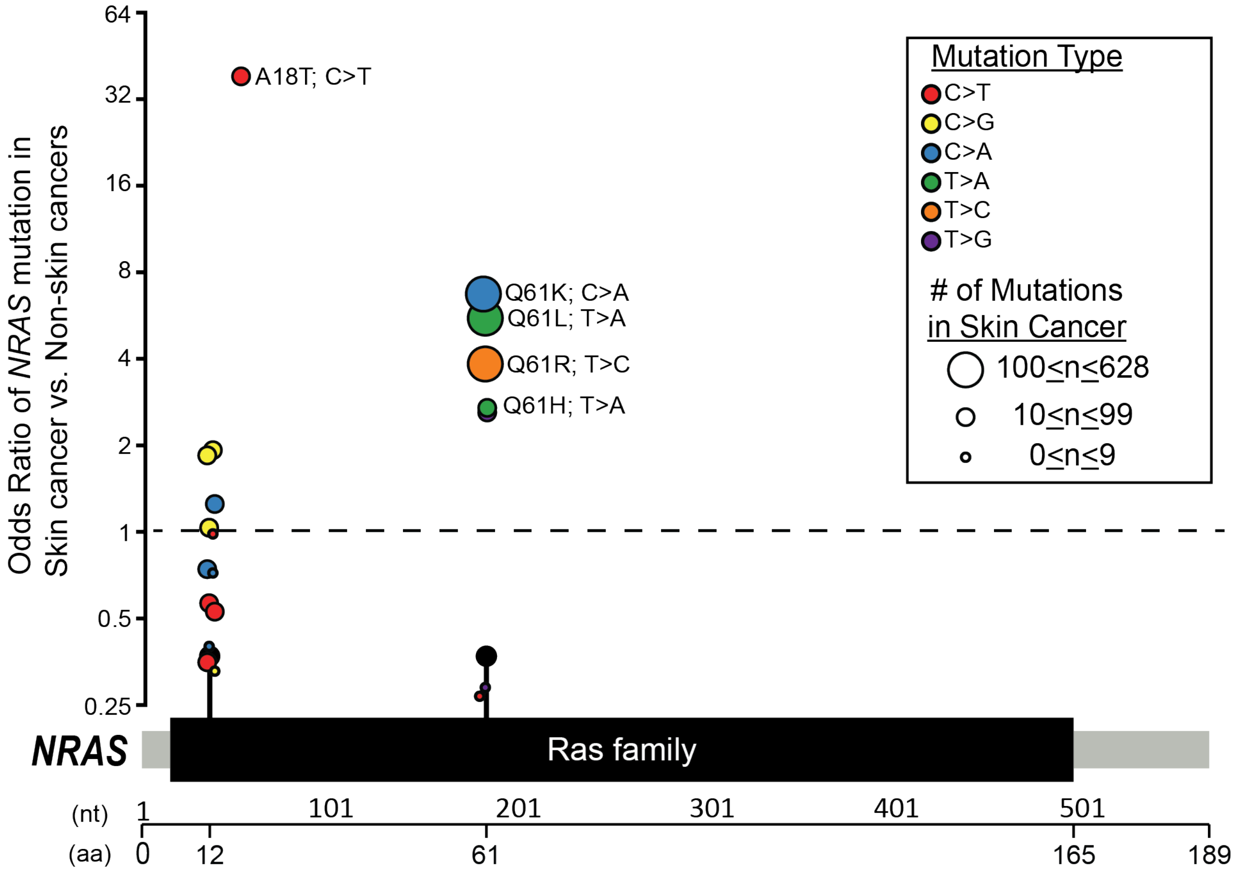

Figure S7: Incidence of recurrent substitution mutations (circles) in the *NRAS* gene stratified between skin and non-skin cancers from the COSMIC database (Tate et al., 2019). Related to Figure 7. Each recurrent mutation is positioned along the x-axis in accordance with its position within the *NRAS* cDNA. Nucleotide (nt) and amino acid (aa) positions are indicated below the schematic of the *NRAS* protein domains. The location of G12 and Q61 hotspot mutations are indicated by lollipop markers. Enrichment for specific recurrent mutations in either skin or non-skin cancers is indicated by odds-ratios of greater than or less than 1 (dashed horizontal line), respectively. Specific substitution types (shown as occurring in the DNA strand containing the pyrimidine base) are color coded and the number of times each recurrent mutation occurs in the dataset is indicated by the size of the circle.

Table S1: Comparison of recurrent *BRAF* mutations in skin and thyroid cancer. Related to Figure 7. Incidence and Odds-Ratios for common recurrent mutations occurring at least 10 times among tumors analyzed within the COSMIC database and originating from either skin or thyroid tissue.

AA Mutation	CDS Mutation	Times Observed in Skin Cancer	Times Observed in Thyroid Cancer	Odds-Ratio in Skin vs Thyroid Cancer
p.V600K	c.1798_1799GT>AA	534	0	infinite
p.V600R	c.1798_1799GT>AG	84	0	infinite
p.D594N	c.1780G>A	19	0	infinite
p.L597S	c.1789_1790CT>TC	16	0	infinite
p.V600D	c.1799_1800TG>AT	13	0	infinite
p.D594G	c.1781A>G	12	0	infinite
p.L597R	c.1790T>G	12	0	infinite
p.L597Q	c.1790T>A	11	0	infinite
p.G466E	c.1397G>A	10	0	infinite
p.V600E	c.1799_1800TG>AA	70	4	35.77482415
p.G469R	c.1405G>A	17	1	34.67557909
p.K601E	c.1801A>G	32	56	1.164964887
p.V600E	c.1799T>A	5791	14303	0.767281652
p.V600_K601>E	c.1799_1801delTGA	5	19	0.536292642
negative	negative	16299	32918	1.030922521
Total Samples		23243	47376	

Table S2: Non-canonical UV mutation classes in yeast. Related to Figures 2 and 7.

Base Change	Sequence Context	Transcription Asymmetry	DNA Lesion	Potential Driver Mutations?
T>C	TT & CT	+	CPD or 6-4PP	*NRAS* Q61R
A>T	TA	+	TA photoproduct	*PTEN* I67K
CT>TA<br>CT>TC	CT	+	CPD or 6-4PP	*BRAF* L597S
AC>CT<br>AC>TT	AC	+	?	*BRAF* V600R<br>*BRAF* V600K

Table S3: Primers used in this study. Related to STAR Methods.

Name	Sequence	Use
OAH152	TTTAGATACTCTTGGCCGTATAACTGGTGTACCAACTGAAAAATCagattgtactgagagtgcac	PCR for rad16Δ fragment
OAH153	TCACTTAAAAACTCCGAGAATAATATATAATAAGAGAATAAAATActgtgcggtatttcacaccg	PCR for rad16Δ fragment
OMP026	ATGGAAGATAAAGAGCAGCAAGACAATGCGAAACTTGAAAACAATGAGTCCTGTGCGGTAT TTCACACCG	PCR for rad26Δ fragment
OMP027	TCATGAAGCATTGTTATTCCTAAATTCTTCATCAAGCACCCACCCTTTACAGATTGTACTGA GAGTGCAC	PCR for rad26Δ fragment
OWY399	AGGCATTATCCGCCAAGTACAATTTTTTACTCTTCGAAGACAGAGTATTTGCTGACATTGGT AATACAGTCAAATTGCAGTACTCTGCGG	ura3 K93V mutation
OWY400	CCGCAGAGTACTGCAATTTGACTGTATTACCAATGTCAGCAAATACTCTGTCTTCGAAGAGT AAAAAATTGTACTTGGCGGATAATGCCT	ura3 K93V mutation
SAR353	CTTAGCATCCCTTCCCTTTG	*URA3* amplification and sequencing
SAR354	GAAGAACGAAGGAAGGAGCACA	*URA3* amplification and sequencing
oHSM002	GATCTGGTGTAGATATCATCGAATGTTTTAGAGCTAG	trp5 E50V gRNA
oHSM003	CTAGCTCTAAAACATTCGATGATATCTACACCA	trp5 E50V gRNA
OHSM001	CTATTCTCAAGGGTTTCCAGGATGGTGGTGTAGATATCATCGTGTTAGGTATGCCCTTCTCT GATCCAATTGCAGATGGTCCTACAATTC	trp5 E50V template
OHSM005	GCACACCGACAGACCATGTCA	*TRP5* amplification and sequencing
OHSM006	CCAGCCTTGGCAGCGTCC	*TRP5* amplification
OWY395	GCGTGTGCACGTATATATATACGCGCGTGTG	In Vitro TA photoproduct substrate
OWY396	Biotin-CACACGCGCGTATATATATACGTGCACACGC	In Vitro TA photoproduct substrate
OSS27	GCGTGTGCACG	TA photoproduct marker: 11 bases
OSS28	GCGTGTGCACGTA	TA photoproduct marker: 13 bases
OSS29	GCGTGTGCACGTATA	TA photoproduct marker: 15 bases
OSS30	GCGTGTGCACGTATATA	TA photoproduct marker: 17 bases
OSS31	GCGTGTGCACGTATATATA	TA photoproduct marker: 19 bases
OSS32	GCGTGTGCACGTATATATATA	TA photoproduct marker: 21 bases
trP1-top	CCTCTCTATGGGCAGTCGGTGAT-phosphorothioate-T	first adapter ligation for UVDE-seq
trP1-bottom	phosphate-ATCACCGACTGCCCATAGAGAGGC-dideoxy	first adapter ligation for UVDE-seq
A1-top	phosphate-ATCCTCTTCTGAGTCGGAGACACGCAGGGATGAGATGGC-dideoxy	Second adapter ligation barcode set 1
A1-bottom	biotin-CCATCTCATCCCTGCGTGTCTCCGACTCAGAAGAGGATNNNNNN-C3 phosphoramidite	Second adapter ligation barcode set 1
A2-top	phosphate-ATCACGAACTGAGTCGGAGACACGCAGGGATGAGATGGC-dideoxy	Second adapter ligation barcode set 2
A2-bottom	biotin-CCATCTCATCCCTGCGTGTCTCCGACTCAGTTCGTGATNNNNNN-C3 phosphoramidite	Second adapter ligation barcode set 2
A3-top	phosphate-ATCTCAGGCTGAGTCGGAGACACGCAGGGATGAGATGGC-dideoxy	Second adapter ligation barcode set 3
A3-bottom	biotin-CCATCTCATCCCTGCGTGTCTCCGACTCAGCCTGAGATNNNNNN-C3 phosphoramidite	Second adapter ligation barcode set 3
A4-top	phosphate-ATCGCGATCTGAGTCGGAGACACGCAGGGATGAGATGGC-dideoxy	Second adapter ligation barcode set 4
A4-bottom	biotin-CCATCTCATCCCTGCGTGTCTCCGACTCAGATCGCGATNNNNNN-C3 phosphoramidite	Second adapter ligation barcode set 4
Primer A	CCATCTCATCCCTGCGTGTCTCCGAC	PCR confirmation and UVDE-seq library amplification
Primer trP1	CCTCTCTATGGGCAGTCGGTGATT	PCR confirmation and UVDE-seq library amplification
CY3-Primer A	CY3-CCATCTCATCCCTGCGTGTCTCCGAC	PCR confirmation of UVDE-seq library amplification
